# Competition between anthocyanin and kaempferol glycosides biosynthesis affects pollen tube growth and seed set of *Malus*

**DOI:** 10.1038/s41438-021-00609-9

**Published:** 2021-08-01

**Authors:** Weifeng Chen, Zhengcao Xiao, Yule Wang, Jinxiao Wang, Rui Zhai, Kui Lin-Wang, Richard Espley, Fengwang Ma, Pengmin Li

**Affiliations:** 1 State Key Laboratory of Crop Stress Biology for Arid Areas/Shaanxi Key Laboratory of Apple, College of Horticulture, Northwest A&F University, Yangling, Shaanxi 712100 China; 2 College of Food Science and Technology, Northwest University, Xi’an, Shaanxi 710069 China; 3 The New Zealand Institute for Plant and Food Research Ltd, Private Bag, 92169 Auckland, New Zealand

**Keywords:** Plant physiology, Metabolomics

## Abstract

Flavonoids play important roles in regulating plant growth and development. In this study, three kaempferol 3-*O*-glycosides were identified and mainly accumulated in flowers but not in leaves or fruits of *Malus*. In *Malus*, flower petal color is normally white, but some genotypes have red flowers containing anthocyanin. Anthocyanin biosynthesis appears to be in competition with kaempferol 3-*O*-glycosides production and controlled by the biosynthetic genes. The white flower *Malus* genotypes had better-developed seeds than the red flower genotypes. In flowers, the overexpression of *MYB10* in *Malus domestica* enhanced the accumulation of anthocyanin, but decreased that of kaempferol 3-*O*-glycosides. After pollination the transgenic plants showed slower pollen tube growth and fewer developed seeds. Exogenous application of different flavonoid compounds suggested that kaempferol 3-*O*-glycosides, especially kaempferol 3-*O*-rhamnoside, regulated pollen tube growth and seed set rather than cyanidin or quercetin 3-*O*-glycosides. It was found that kaempferol 3-*O*-rhamnoside might regulate pollen tube growth through effects on auxin, the Rho of plants (ROP) GTPases, calcium and the phosphoinositides signaling pathway. With the inhibition of auxin transport, the transcription levels of Heat Shock Proteins (HSPs) and ROP GTPases were downregulated while the levels were not changed or even enhanced when blocking calcium signaling, suggesting that HSPs and ROP GTPases were downstream of auxin signaling, but upstream of calcium signaling. In summary, kaempferol glycoside concentrations in pistils correlated with auxin transport, the transcription of HSPs and ROP GTPases, and calcium signaling in pollen tubes, culminating in changes to pollen tube growth and seed set.

## Introduction

Flavonoids are common secondary metabolites with more than 8000 forms discovered in plants. The flavonoids contain six major classes including chalcones, flavones, isoflavonoids, flavanones, flavonols, and anthocyanins^1^. Flavonoid biosynthesis begins with the substrate 4-coumaroyl-CoA, which is stepwise catalyzed by chalcone synthase (CHS) and chalcone flavanone isomerase (CHI) to produce naringenin. Naringenin can be hydroxylated at the 3′-position of the B-ring by flavonoid 3′-hydroxylase (F3′H) to produce eriodictyol or hydroxylated at the 3-position of the C-ring by flavanone 3-hydroxylase (F3H) to produce dihydrokaempferol. Eriodictyol or dihydrokaempferol can be further hydroxylated at the 3-position of the C-ring or at the 3′-position of the B-ring by F3H or F3′H to produce dihydroquercetin^2,3^. In some plants, dihydrokaempferol can also be hydroxylated at both the 3′- and 5′-positions of the B-ring by flavonoid 3′5′-hydroxylase to produce dihydromyricetin^4,5^. Dihydrokaempferol, dihydroquercetin, and dihydromyricetin are catalyzed by flavonol synthase (FLS) to produce kaempferol, quercetin, and myricetin, respectively, or they are catalyzed by dihydroflavonol 4-reductase (DFR) and anthocyanin synthase (ANS) to produce anthocyanidins^4–7^. Since the aglycones (kaempferol, quercetin, myricetin, and anthocyanidin) are not stable in plants, they are usually glycosylated by the catalysis of UDP-glycose: flavonoid glycosyltransferase (UGT) and then transported and stored in vacuoles. This suggests that there is likely to be competition for the same substrates for the synthesis of different kinds of flavonoid end products^8–10^. For some plants, the variation in flower color is determined by competition between the synthesis of flavonols and anthocyanins^7,11^.

Besides affecting color, flavonoids have other important biological functions in plants. The inhibition of flavonoid synthesis by silencing *MdCHS* or *MdUGT88F1* in apple plants resulted in shortened internode lengths, smaller leaves, and a greatly reduced growth rate^12,13^. In the flowers of *Petunia*, the UV absorbance due to the presence of flavonols plays an important role in attracting pollinators with corresponding flower signal preferences^7^. It was also found that flavonols can modulate stomata aperture behavior^14,15^, root development and gravitropism^16–18^, leaf and trichome development in *Arabidopsis*^19,20^, self-compatibility in *Brassicaceae*^21^, and protect plants against pathogens^22^.

Recently, Muhlemann et al. reported that flavonol might control pollen tube growth and integrity by regulating ROS homeostasis in tomato under high-temperature stress^23^. The *anthocyanin reduced (are)* tomato mutant, which lacks a functional F3H enzyme, has reduced flavonol accumulation in pollen grains and tubes, and produced fewer pollen grains as well as having impaired pollen viability, germination, tube growth, and integrity resulting in reduced seed set^23^. The mutation of *F3H* might also decrease the flavonol and anthocyanin accumulation in other flower tissues such as the pistil. However, whether the flavonols in the pistil affected pollen germination and tube growth was not clarified. Moreover, the precise nature of the flavonol compounds in pollen that are involved in this regulation of pollen development was not confirmed.

Of the numerous variety of flavonol compounds in plants, the common major flavonol aglycones are quercetin and kaempferol, with their derivatives being more abundant. The derivatives of these different aglycones might play different roles in plants. For instance, quercetin rather than kaempferol derivatives were shown to modulate basipetal root auxin transport, gravitropism, and elongation in *Arabidopsis*^18^. In addition, for any flavonol aglycone the different types of UDP-sugars and the position and amount of glycosylation, the glycosylated version might show different functions. For example, it was found that it was specifically kaempferol 3-*O*-rhamnoside-7-*O*-rhamnoside, as opposed to any other kaempferol glycoside compounds, that acted as an endogenous polar auxin transport inhibitor in *Arabidopsis* shoots^24^. Phloretin and its derivatives, a unique group of flavonoids in *Malus*, showed different antioxidant capacities, even when the chemical structures of each compound simply differed in one hydroxyl group or glycosylation^25,26^. So, to clarify the precise relationships between flavonoid structures and their physiological functions on the pollens, further studies are required.

For *Malus*, domesticated apple cultivars provide fresh apple fruits or juice for consumption, whereas ornamental crabapple cultivars are widely used in landscaping for their colorful flowers and, largely inedible, fruits. Both the domestic and ornamental cultivars originally derive from the wild species of *Malus* through different routes of domestication, and the different genotypes of wild species come from the evolution of *Malus*^27,28^. The evolution process differs from domestication. For instance, the traits considered beneficial by human beings such as fruit size, texture, flavor, and other fruit quality-related traits of *Malus*, were selected for improvement during domestication^28,29^, whereas the positive traits that contribute to plant survival are likely to be retained and enhanced during *Malus* evolution. Although positive traits that contribute to plant survival might be lost or abated during domestication, this does not necessarily compromise the cultivar. For instance, in some cultivars, positive traits related to seed development may be lost, but reproduction is still feasible through grafting or other asexual ways. However, for wild species that need to survive in natural conditions, long-term survival may be threatened under the pressure of natural selection if such traits were lost.

To date, a large number of *Malus* ornamental or edible red flower/flesh cultivars have been successfully developed or are being developed in breeding programs. However, for wild species of *Malus*, the majority of them have white flowers/flesh (some with pink bud). So, the question arises as to whether flower color is in some way disadvantageous and/or relates to the reproductive capacity of *Malus*. This is possibly why the wild species of *Malus* seldom have red flowers. In this study, we used different flower color genotypes of *Malus* to investigate the relationship between flower color and seed set, an important trait for sexual reproduction capacity. In particular, we focused on the association of flavonoid metabolism in pistils and on pollen tube growth and seed set.

## Results

### Correlation between flower color and seed set

**Fig. 1:**
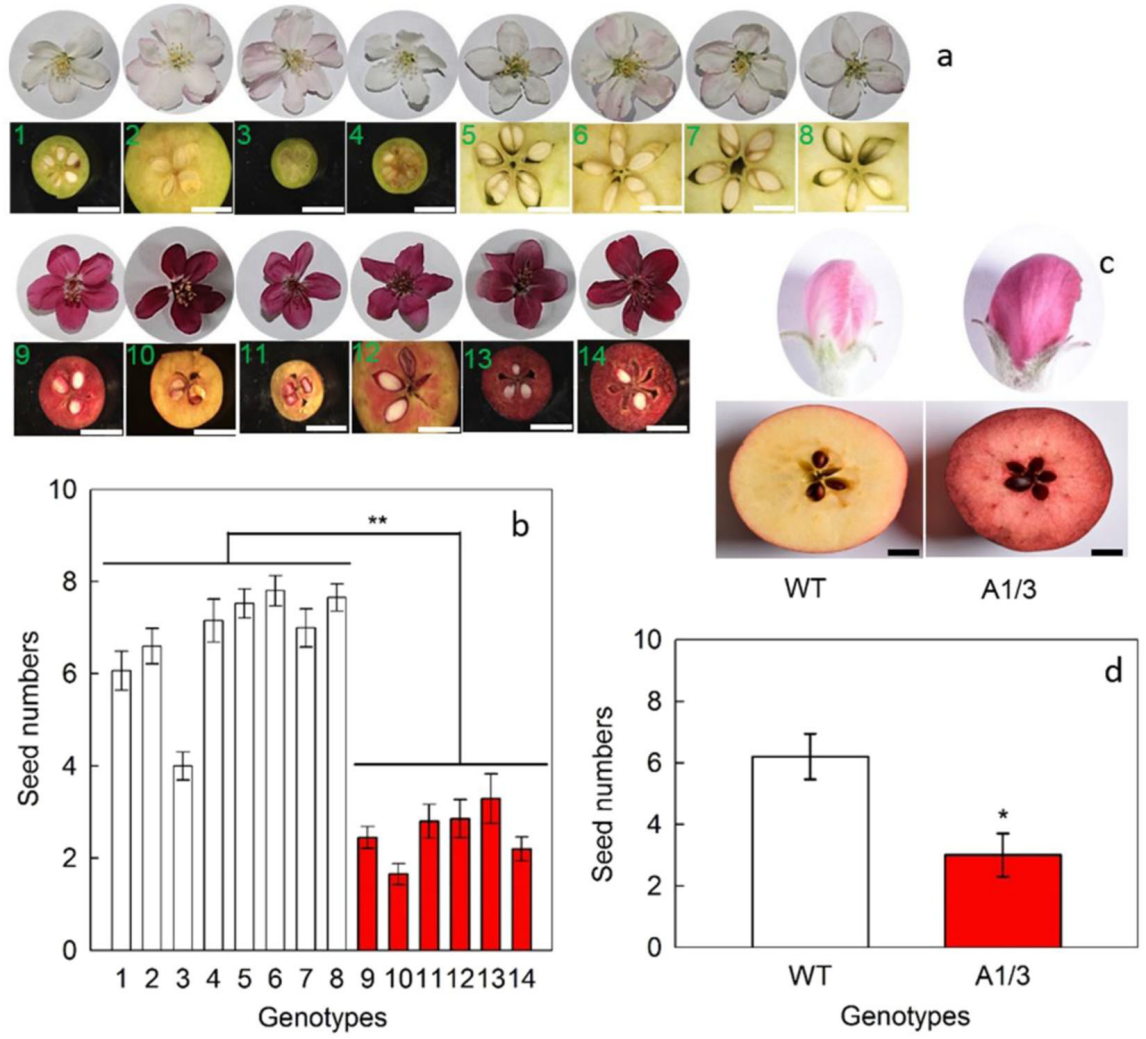
The phenotypes and seed numbers of different genotypes of Malus plants. **a**, **b** White flower genotypes: 1, *M. hupehensis*; 2, *M. micromalus*; 3, *M. halliana*; 4, *M. baccata*; 5, *M. domestica* ‘Golden Delicious’; 6, *M. domestica* ‘Fuji’; 7, *M. domestica* ‘Red Delicious’; 8, *M. domestica* ‘Gala’; and red flower genotypes; 9*, M*. ‘Sparkler’; 10, *M*. ‘Radiant’; 11, *M*. ‘Adams’; 12, *M*. ‘Kelsey’; 13, *M*. ‘Perfect Purple’; 14, *M*. ‘Royalty’. **c**, **d** WT, wild type of *M. domestica* ‘Royal Gala’; A1/3, transgenic line with *MdMYB10* overexpression of ‘Royal Gala’. **a**, **c** Bars = 1 cm. **b**, **d** White bars represent white flower genotypes and red bars represent red flower genotypes; data are presented as mean ± SE (*n* = 5); “*” and “**” mean significant difference between the white flower and the red flower genotypes at *P* < 0.05 and at *P* < 0.01, respectively, *t*-test

Fourteen genotypes of *Malus*, including eight white (pink) flower (WF) and six red flower (RF) genotypes, were collected to assess the seed set of fruits (Fig. 1a). Interestingly, it was found that for WF genotypes all seed carpels were full, while for RF genotypes some empty seed carpels were found. Moreover, the numbers of well-developed seeds were significantly fewer in RF genotypes than that in WF genotypes (Fig. 1b). In previous studies, it was shown that overexpression of *MYB10*, a key transcript factor that regulates anthocyanin synthesis in apple, could strongly elevate the anthocyanin content of apple plants^30,31^. In this study, the transgenic apple plants with *MYB10* overexpression (RF) and its wild type *M. domestica* ‘Royal Gala’ (WF), was also used to test the correlation between flower color and seed set. It was found that the transgenic fruits had significantly fewer seeds compared with the wild type (WT) (Fig. 1c, d).

### Identification of kaempferol 3-*O*-glycosides

**Fig. 2:**
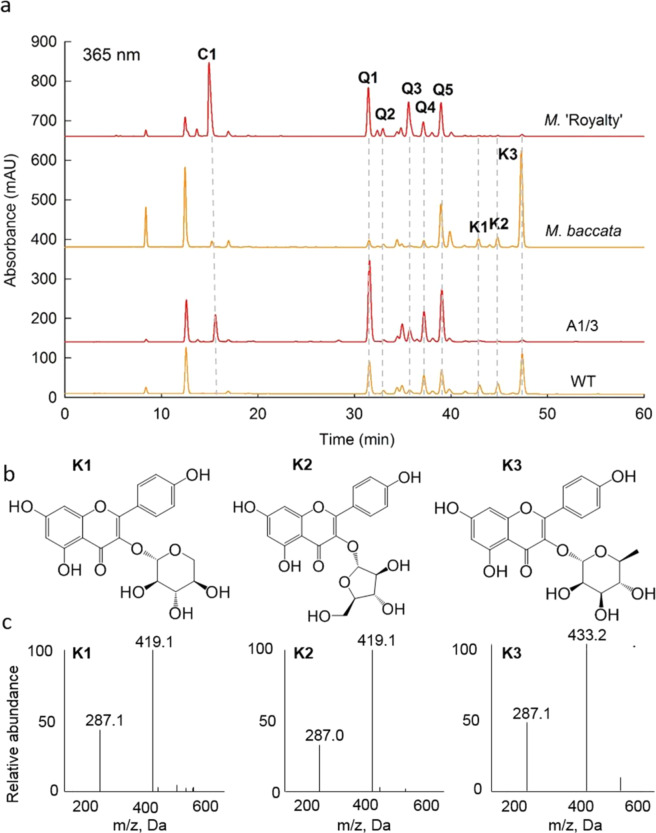
The chromatogram of flavonoids in petals of *Malus* and the identification of kaempferol glycosides. **a** The HPLC chromatogram of flavonoids detected at 365 nm (*M. baccata*, white flower genotype; *M*. ‘Royalty’, red flower genotype; WT, *M. domestica* ‘Royal Gala’; A1/3, the *MYB10* overexpressed transgenic line of ‘Royal Gala’). **b** Structures of kaempferol glycosides. **c** ESI-MS spectra of three kaempferol glycosides at positive mode. C1: cyanidin 3-*O*-galactoside; Q1: quercetin 3-*O*-galactoside; Q2: quercetin 3-*O*-glucoside; Q3: quercetin 3-*O*-xyloside; Q4: quercetin 3-*O*-arabinoside; Q5: quercetin 3-*O*-rhamnoside; K1, kaempferol 3-*O*-xyloside; K2, kaempferol 3-*O*-arabinoside, and K3, kaempferol 3-*O*-rhamnoside

The chromatograms of flower flavonoids showed that the transgenic lines (A1/3, A4) of ‘Royal Gala’ accumulated more anthocyanin (cyanidin 3-*O*-galactoside), but less of the three unknown flavonoid compounds (K1, K2, and K3) compared with the WT (Fig. 2a). Similarly, the RF genotypes also showed significantly lower amounts of the three unknown compounds compared with the WF genotypes.

To identify the three unknown flavonoid compounds, 5 kg of *M*. *domestica* ‘Fuji’ flowers was used to isolate the compounds with a purity higher than 98%, and then ESI-MS, ^1^H NMR, and ^13^C NMR were used to analyze the structures of the three compounds (Fig. 2b, c, Table S1, Fig. S1).

The ESI-MS analysis showed that both K1 and K2 had a quasi-molecule ion at *m/z* 419.1 [M + H]^+^, while K3 had a quasi-molecule ion at *m/z* 433.2 [M + H]^+^. However, all of them had a fragment at *m/z* 287.1, consistent with the molecular weight of kaempferol in positive mode (Fig. 2c). The fragments indicate a loss of a pentoside moiety for K1 and K2, and a loss of the rhamnoside moiety for K3. Combined with the ^1^H and ^13^C NMR analysis results (Table S1), K1, K2 and K3 were identified as kaempferol 3-*O*-β-xylopyranoside (kaempferol 3-*O*-xyloside), kaempferol 3-*O*-α-arabinofuranoside (kaempferol 3-*O*-arabinoside) and kaempferol 3-*O*-α-rhamnopyranoside (kaempferol 3-*O*-rhamnoside), respectively.

### Correlation between cyanidin 3-*O*-glycosides and kaempferol 3-*O*-glycosides synthesis in *Malus*

**Fig. 3:**
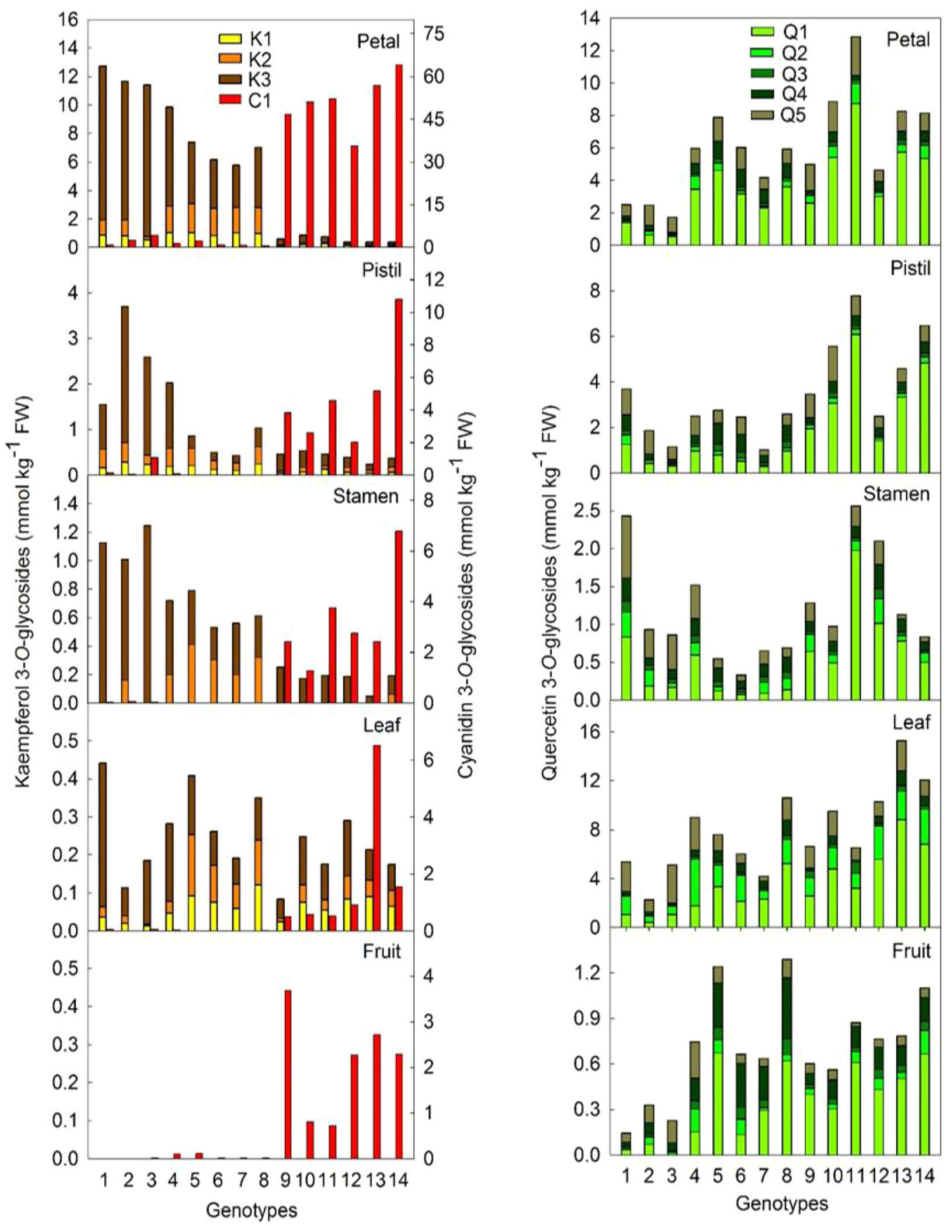
Flavonoid compounds concentration in different tissues of *Malus* germplasm resources. 1, *M. hupehensis*; 2, *M. micromalus*; 3, *M. halliana*; 4, *M. baccata*; 5, *M. domestica* ‘Golden Delicious’; 6, *M. domestica* ‘Fuji’; 7, *M. domestica* ‘Red Delicious’; 8, *M. domestica* ‘Gala’; and red flower genotypes: 9*, M*. ‘Sparkler’; 10, *M*. ‘Radiant’; 11, *M*. ‘Adams’; 12, *M*. ‘Kelsey’; 13, *M*. ‘Perfect Purple’; 14, *M*. ‘Royalty’. C1: cyanidin 3-*O*-galactoside; K1, kaempferol 3-*O*-xyloside; K2, kaempferol 3-*O*-arabinoside; K3, kaempferol 3-*O*-rhamnoside; Q1: quercetin 3-*O*-galactoside; Q2: quercetin 3-*O*-glucoside; Q3: quercetin 3-*O*-xyloside; Q4: quercetin 3-*O*-arabinoside; and Q5: quercetin 3-*O*-rhamnoside. The flower petals were collected at stage 1 as described in the materials and methods. Data are presented as the average of five replicates

To investigate whether there is any relationship between anthocyanin and kaempferol glycosides, flavonoids compounds in different tissues of WF and RF were quantified. The quantitative analysis of kaempferol 3-*O*-glycosides showed that kaempferol 3-*O*-rhamnoside was the major kaempferol 3-*O*-glycosides compound in *Malus* (Fig. 3). Moreover, kaempferol 3-*O*-glycosides mainly accumulated in flowers and very little in leaves and fruits. It was also found that kaempferol 3-*O*-glycosides did not accumulate in pollen, although they accumulated in pollen sacs (Fig. S2). Further analysis showed that the concentrations of kaempferol 3-*O*-glycosides had a significantly negative correlation with that of cyanidin 3-*O*-glycosides and quercetin 3-*O*-glycosides (quercetin 3-*O*-galactoside, quercetin 3-*O*-glucoside, quercetin 3-*O*-xyloside, quercetin 3-*O*-arabinoside, and quercetin 3-*O*-rhamnoside) in flowers (Fig. S3), but not in leaf and fruit (Fig. 3). Moreover, the concentrations of kaempferol 3-*O*-glycosides in flowers were significantly higher whereas that of cyanidin 3-*O*-glycosides and quercetin 3-*O*-glycosides were significantly lower in WF compared with RF genotypes (Fig. 3, Fig. S4). No significant difference was found in dihydrochalcone (phlorizin, trilobatin, and sieboldin) concentrations between WF and RF genotypes (Fig. S5).

**Fig. 4:**
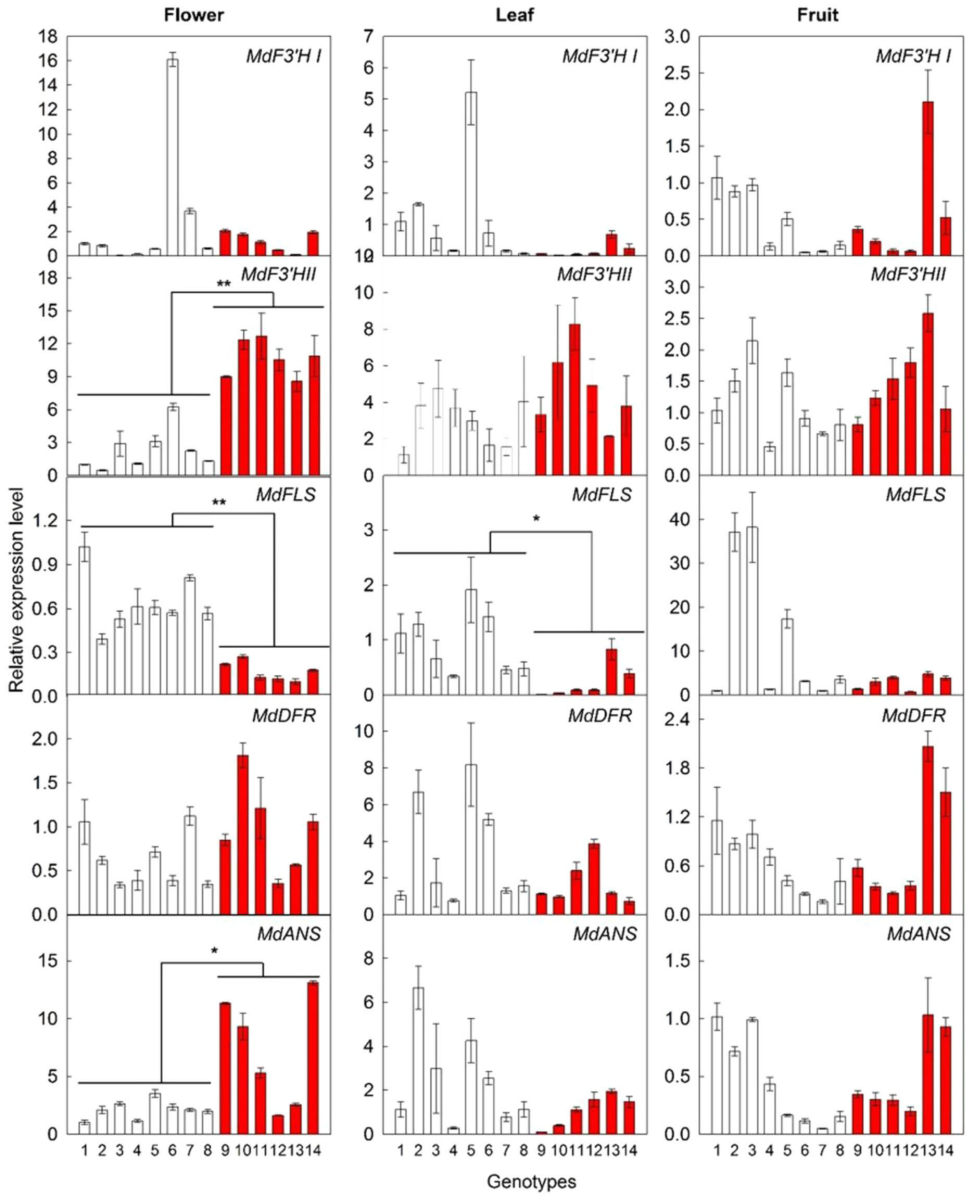
Expressions of key genes involved in flavonoids synthesis in different tissues of *Malus* germplasm resources. 1, *M. hupehensis*; 2, *M. micromalus*; 3, *M. halliana*; 4, *M. baccata*; 5, *M. domestica* ‘Golden Delicious’; 6, *M. domestica* ‘Fuji’; 7, *M. domestica* ‘Red Delicious’; 8, *M. domestica* ‘Gala’; and red flower genotypes: 9*, M*. ‘Sparkler’; 10, *M*. ‘Radiant’; 11, *M*. ‘Adams’; 12, *M*. ‘Kelsey’; 13, *M*. ‘Perfect Purple’; 14, *M*. ‘Royalty’. F3′H, flavonoid 3′-hydroxylase; FLS, flavonol synthase; DFR dihydroflavonol 4-reductase; ANS, anthocyanin synthase. White bars represent white flower genotypes and red bars represent red flower genotypes. The flower petals were collected at stage 1 as described in the materials and methods. Data are presented as mean ± SE (*n* = 3); “*” and “**” mean significant difference between the white flower and the red flower genotypes at *P* < 0.05 and *P* < 0.01, respectively, *t*-test

**Fig. 5:**
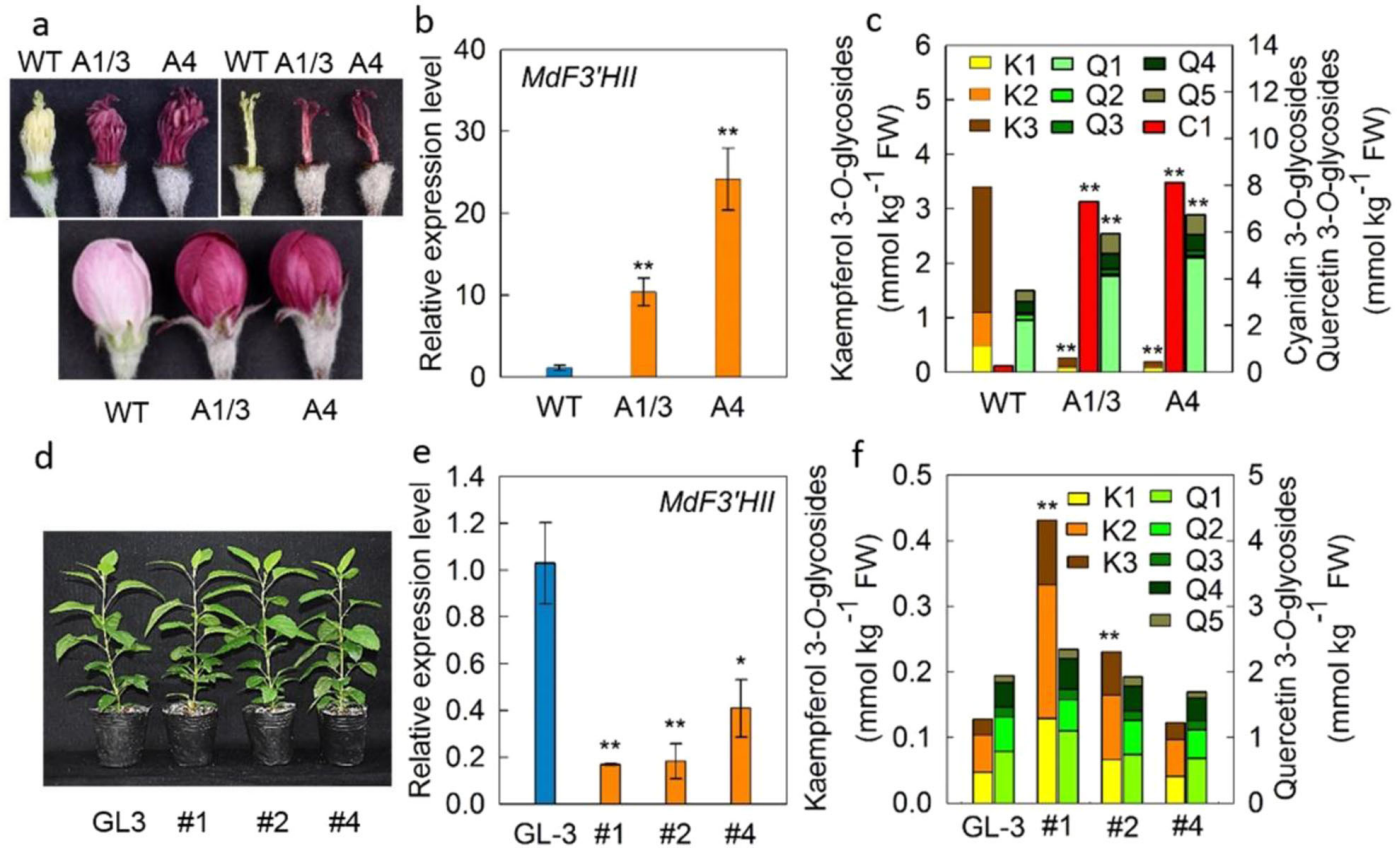
The phenotypes, gene expression, and flavonoid compound concentrations in transgenic apple plants with *MdMYB10* overexpression or the RNAi of *MdF3*′*HII*. **a**–**c** the flower phenotypes, the expression of *MdF3*′*HII* and the concentrations of flavonoid compounds in the flowers of *M. domestica* ‘Royal Gala’ (WT) and its transgenic lines (A1/3, A4) with *MdMYB10* overexpression; **d**–**f** the seedling phenotypes, the expression of *MdF3*′*HII* and the concentrations of flavonoid compounds in the leaves of *M. domestica* ‘GL3’ (GL-3) and its transgenic lines (#1, #2, #4) with the RNAi of *MdF3*′*HII*. C1: cyanidin 3-*O*-galactoside; K1, kaempferol 3-*O*-xyloside; K2, kaempferol 3-*O*-arabinoside, K3, kaempferol 3-*O*-rhamnoside; Q1: quercetin 3-*O*-galactoside; Q2: quercetin 3-*O*-glucoside; Q3: quercetin 3-*O*-xyloside; Q4: quercetin 3-*O*-arabinoside; and Q5: quercetin 3-*O*-rhamnoside. **b**, **c**, **e**, **f** Data are presented as mean ± SE (*n* = 3 for **b** and **e**, *n* = 5 for **c** and **f**); “*” and “**” mean significant difference between the wild type and the transgenic line at *P* <0.05 and *P* < 0.01, respectively, *t*-test

The synthesis of kaempferol competes with that of cyanidin for the same substrates, naringenin or dihydrokaempferol (Fig. S6). To explore the negative correlation between kaempferol 3-*O*-glycosides and cyanidin 3-*O*-glycosides in flowers, the expression of key genes involved in the kaempferol and cyanidin 3-*O*-glycosides synthesis pathway (Fig. S6), including *MdF3*′*HI*, *MdF3*′*HII*, *MdFLS*, *MdDFR*, and *MdANS*, were assayed. Among these five genes, *MdF3*′*HI* did not show any difference between WF and RF genotypes (Fig. 4, Fig. S7). However, *MdF3*′*HII* and *MdFLS* showed a significant difference between WF and RF genotypes before full bloom. The expression level of *MdF3*′*HII* was significantly lower, but that of *MdFLS* was significantly higher in the flowers of WF genotypes compared with that in RF genotypes. No significant difference was found between WF and RF genotypes in the transcript abundance of *MdF3*′*HII* in leaf or fruit, while *MdFLS* showed higher expression levels in leaves of WF compared with that of RF. Before full bloom, the expression levels of *MdDFR* and *MdANS* were higher in the flowers of RF, but similar in leaf and fruit compared with that of WF (Fig. 4, Fig. S7). The *MYB10*-overexpressed transgenic flowers of ‘Royal Gala’ showed significant increases in the expression levels of *MdF3*′*HII*, but significant decreases in the levels of *MdFLS* compared with the WT (Fig. 5a, b, Fig. S8a). Moreover, the transgenic flowers accumulated more cyanidin 3-*O*-galactoside, but less kaempferol 3-*O*-glycosides (Fig. 5c). Concentrations of dihydrochalcones were comparable in the transgenic and the WT flowers (Fig. S8b).

An RNA interference (RNAi) approach was used to repress the expression of *MdF3*′*HII* in ‘GL3’ apple seedlings and three transgenic lines were obtained (Fig. 5d). The transformation of *MdF3*′*HII* did not affect the expression level of *MdFLS* (Fig. S8c). Among the three transgenic lines of ‘GL3’, two lines showed significantly higher concentrations of kaempferol 3-*O*-glycosides in the leaves compared with that in the WT. Meanwhile, different from that in the flowers of WF genotypes, the fraction of kaempferol 3-*O*-rhamnoside to total kaempferol 3-*O*-glycosides was reduced in the transgenic ‘GL3’ leaves (Fig. 5f). The concentration of other flavonoids such as quercetin 3-*O*-glycosides and dihydrochalcones remained unchanged in the transgenic leaves (Fig. 5, Fig. S8d).

### Effects of flavonoids on pollen tube growth and seed set

**Fig. 6:**
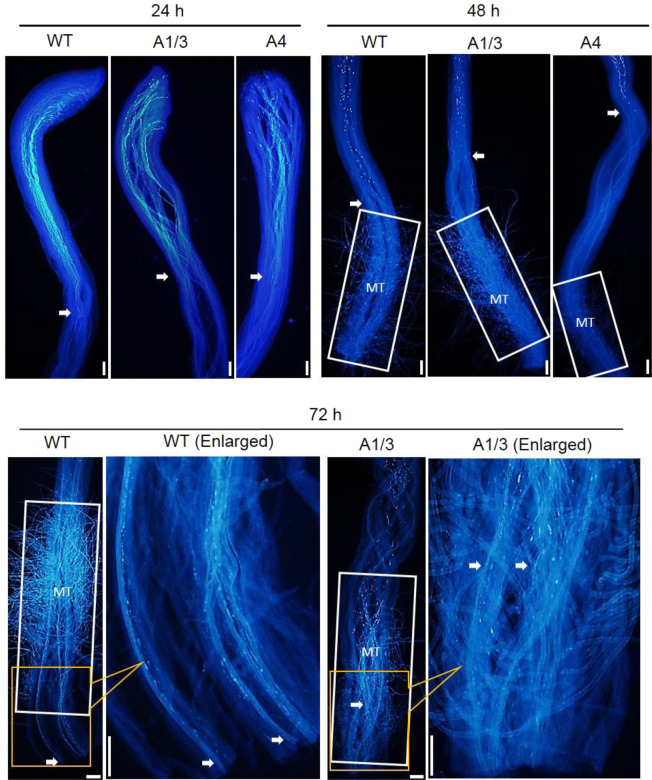
The tube growth of *Malus domestica* ‘Granny Smith’ pollen in flowers of *M. domestica* ‘Royal Gala’ and its transgenic lines with *MdMYB10* overexpression. The stained pollen tubes were observed with a fluorescence microscope at 24, 48, and 72 h after pollination. WT, wild type; A1/3 and A4, transgenic lines. The white arrows indicate the locations where pollen tubes arrived; MT: multicellular trichome, Bars = 50 μm

To investigate whether the flavonoids in pistils affect pollen tube growth in vivo, the *MYB10*-overexpressed transgenic flowers as well as its WT were pollinated with the pollen grains of ‘Granny Smith’. After pollination, it was found that the tube growth of the pollen grains of ‘Granny Smith’ was significantly slower in the *MYB10*-overexpressed transgenic flowers compared with that in the WT (Fig. 6). At 48 h after pollination, the pollen tube had arrived at the style region with multicellular trichomes in the WT, but this was not the case in the transgenic flowers. At 72 h, the pollen tube had passed through the end of the style in the WT, but not in the transgenic flowers.

**Fig. 7:**
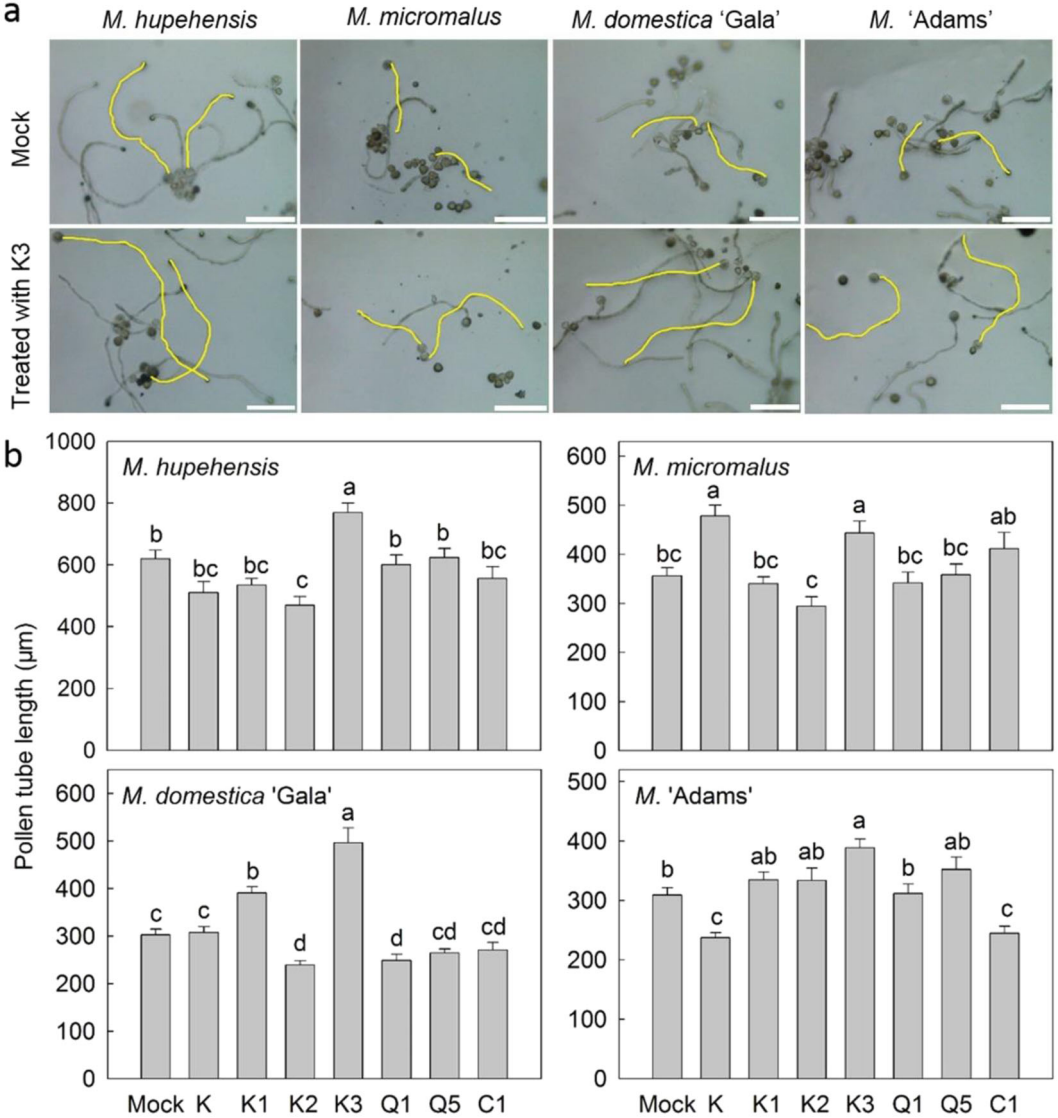
Effects of exogenous applications of flavonoids on pollen tube growth of *Malus*. K, kaempferol; K1, kaempferol 3-*O*-xyloside; K2, kaempferol 3-*O*-arabinoside; K3, kaempferol 3-*O*-rhamnoside; Q1, quercetin 3-*O*-galactoside; Q5, quercetin 3-*O*-rhamnoside; C1, cyanidin 3-*O*-galactoside. **a** Bars = 200 μm. **b** Data are presented as mean ± SE (*n* = 5); different letters above the bars indicate statistically significant differences at *P* < 0.01, LSD

**Fig. 8:**
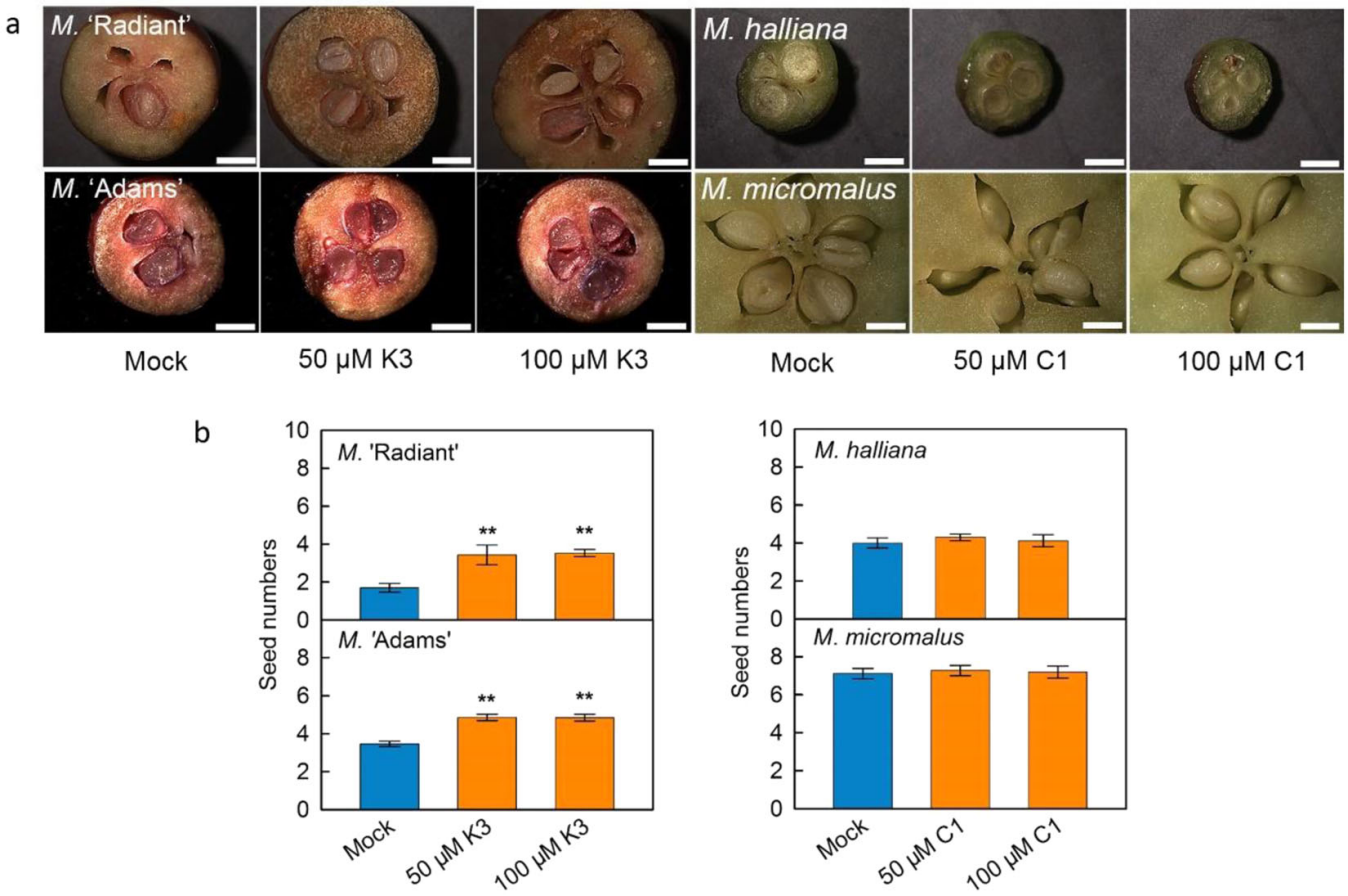
Effect of exogenous applications of flavonoids on seed set of *Malus*. Red flower genotypes, *M*. ‘Radiant’ and *M*. ‘Adams’; white flower genotypes, *M. halliana* and *M. micromalus*. K3, kaempferol 3-*O*-rhamnoside; C1, cyanidin 3-*O*-galactoside. **a** Bars = 2 mm. **b** Data are presented as mean ± SE (*n* = 5); “**” above the bars indicate statistically significant differences between mock and treatment at *P* < 0.01, *t*-test

As there was no difference in pollen viability (the ability of pollen to complete post-pollination events and to effect fertilization^32^) between WF and RF genotypes (Fig. S9), pollen was treated with different flavonoid compounds in vitro in order to test whether they might affect pollen tube growth (Fig. 7). It was found that quercetin 3-*O*-glycosides and cyanidin 3-*O*-galactoside (the major cyanidin 3-*O*-glycosides in *Malus*) had little effect on pollen tube growth, while kaempferol and kaempferol 3-*O*-glycosides, especially kaempferol 3-*O*-rhamnoside, enhanced the growth four hours after treatment. Moreover, the kaempferol 3-*O*-rhamnoside treatments in vivo significantly increased the amount of well-developed seed for two RF genotypes, *M*. ‘Adams’ and *M*. ‘Radiant’, while cyanidin 3-*O*-galactoside did not affect the amount of well-developed seed for two WF genotypes, *M. micromalus* and *M. halliana* (Fig. 8).

### Transcriptomic analysis of treated pollen

**Fig. 9:**
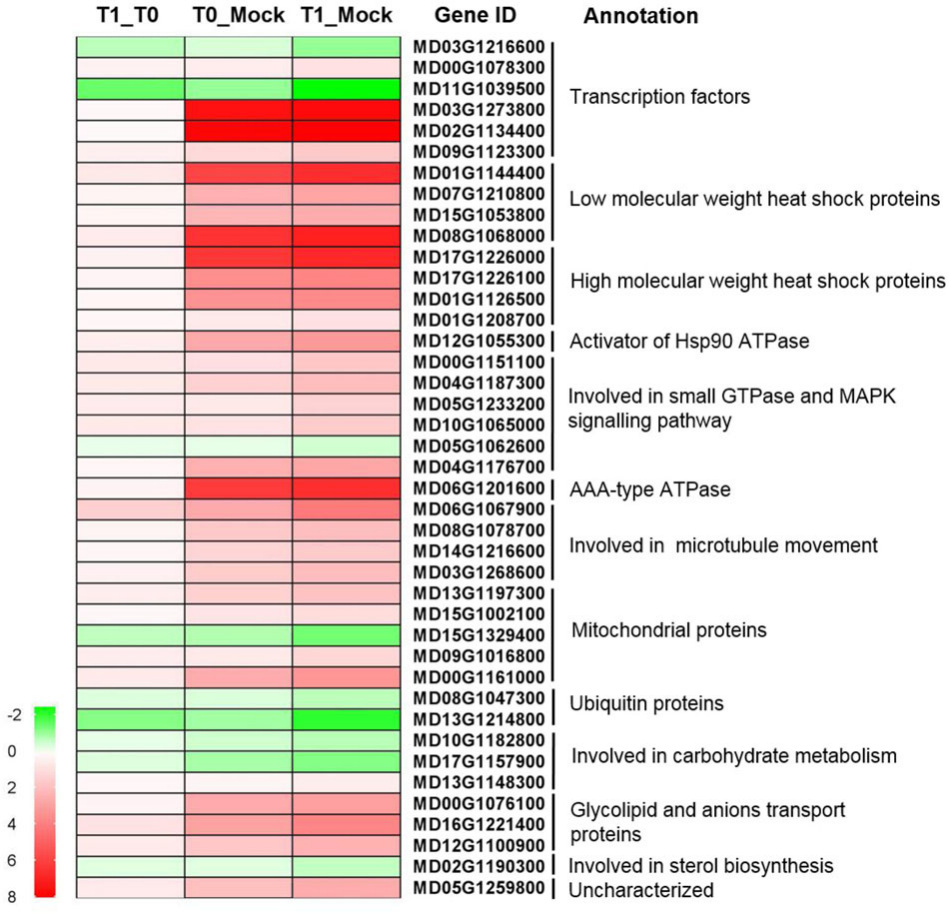
Gene expressions during pollen tube growth after different treatments. Mock: pollen cultured for 0 h, T0: pollen cultured for 4 h, T1: pollen cultured for 4 h with 1 μM kaempferol 3-*O*-rhamnoside treatment

To further investigate the effect of kaempferol 3-*O*-rhamnoside on pollen tube growth, transcriptome analysis was carried out using the pollen (including pollen tubes) treated with and without kaempferol 3-*O*-rhamnoside for 4 h in vitro, with pollen at 0 h being set as control. An intersection was taken among the differentially expressed genes (DEGs) between 4 and 0 h without kaempferol 3-*O*-rhamnoside treatment (T0_Mock), the DEGs between 4 and 0 h with kaempferol 3-*O*-rhamnoside treatment (T1_Mock), and the DEGs between 4 h with and without kaempferol 3-*O*-rhamnoside treatment (T1_T0). The DEGs with similar expression trends were selected. Only 41 DEGs (Log_2_^x^ > 0.2) were found, including 32 upregulated and nine downregulated genes (Fig. 9, Table S2).

Among the DEGs, there were six transcription factors (TF), including two WD40 (MD03G1216600, MD00G1078300), one NAC (MD09G1123300), one TF with BTB/POZ domain (MD11G1039500), and two TFs with histone-fold (MD03G1273800, MD02G1134400). The DEGs also included four low-molecular weight heat shock proteins (HSPs) (MD01G1144400, MD07G1210800, MD15G1053800, MD08G1068000); four high molecular weight HSPs (MD17G1226000, MD17G1226100, MD01G1126500, MD01G1208700); one activator of HSP 90 ATPase (MD12G1055300); one AAA-type ATPase (MD06G1201600); six genes involved in small GTPase and MAPK mediated signal transduction: one rab escort protein (MD10G1065000), one guanine nucleotide exchange factor (MD05G1233200), two serine/threonine protein kinase (MD00G1151100, MD04G1187300), one phosphatidylinositol transfer protein (MD05G1062600), and one phosphatidylinositol-mediated signaling protein (MD04G1176700). There were four genes involved in microtubule-based movement: two kinesin proteins (MD06G1067900, MD08G1078700) and two microtubule-associated movement proteins (MD14G1216600, MD03G1268600); and five mitochondrial genes: two pentatricopeptide repeat superfamily proteins (MD13G1197300, MD15G1002100), one prohibition (MD09G1016800), one mitochondrial inner membrane protein (MD15G1329400), and one synaptotagmin (MD00G1161000). Other DEGs included two ubiquitin genes (MD08G1047300, MD13G1214800); three genes involved in carbohydrate metabolism (MD10G1182800, MD17G1157900, MD13G1148300); three genes involved in glycolipid and anions transport (MD00G1076100, MD16G1221400, MD12G1100900); one gene involved in sterol biosynthesis (MD02G1190300); and one uncharacterized gene (MD05G1259800). The expression of some genes, especially *HSPs* and two genes related to small GTPase, were doubly checked using real-time qPCR (Fig. S10). The qPCR results showed that most of the differences in the gene expressions were consistent with that obtained from transcriptome analysis.

### Effects of auxin and calcium on pollen tube growth

**Fig. 10:**
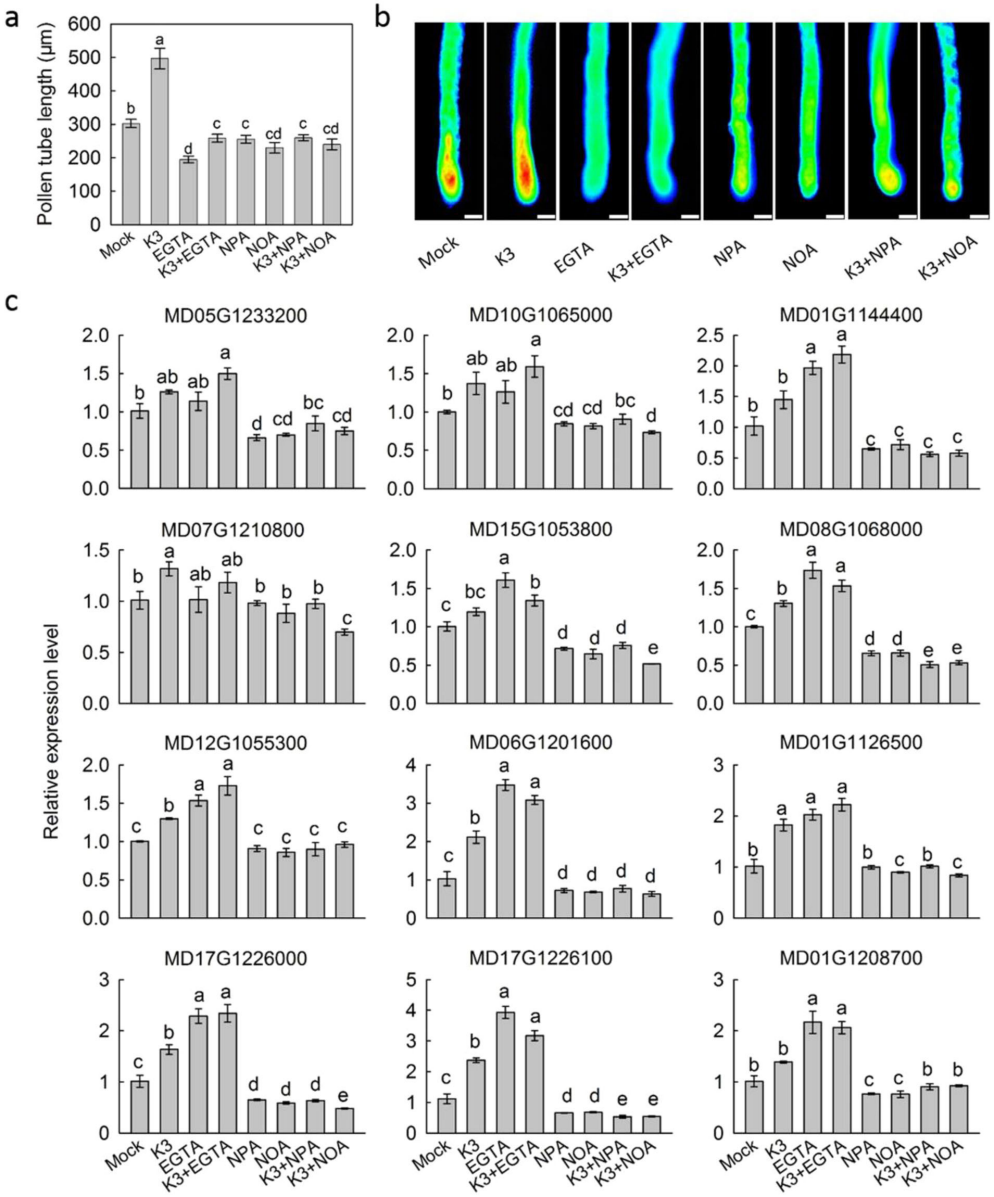
Effect of different chemicals on pollen tube growth. **a** the effect of different chemicals on pollen tube growth rate; **b** the effect of different chemicals on calcium accumulation in pollen tips; **c** the effect of different chemicals on gene expression during pollen tube growth. K3, kaempferol 3-*O*-rhamnoside; EGTA, a chelator of calcium ion; NPA, 1-*N*-naphthylphthalamic acid, an inhibitor of auxin efflux from plant cells; NOA, β-naphthoxyacetic acid, an inhibitor of auxin influx from plant cells. Data are presented as mean ± SE (*n* = 5); different letters above the bars indicate statistically significant differences among treatments at *P* < 0.01, LSD

The application of β-naphthoxyacetic acid (NOA), an inhibitor of auxin influx from plant cells; 1-*N*-naphthylphthalamic acid (NPA), an inhibitor of auxin efflux from plant cells; and EGTA, a chelator of calcium ion, all significantly inhibited pollen tube growth (Fig. 10a). These three chemical treatments also lowered the abundance of calcium ions at the tip of pollen tubes (Fig. 10b). The enhancement of pollen tube growth and calcium ion abundance by kaempferol 3-*O*-rhamnoside treatment also disappeared with the combined NOA, NPA, or EGTA treatment. It was also found that the NOA and NPA treatments lowered the expression levels of genes encoding HSPs and those involved in the small GTPase signaling pathway, while EGTA either did not change or increased their expression (Fig. 10c).

## Discussion

Our present study demonstrated that the biosynthesis of kaempferol 3-*O*-glycosides competed with that of anthocyanin in the flowers of *Malus*, which partly determined the flower color (Figs. 1–5). Moreover, kaempferol 3-*O*-glycosides, especially kaempferol 3-*O*-rhamnoside, was involved in the regulation of pollen tube growth and seed set (Figs. 6–10).

Dihydrochalcones, cyandin 3-*O*-galactoside, kaempferol 3-*O*-glycosides, and quercetin 3-*O*-glycosides were the major flavonoid compounds detected in the flowers of *Malus*. As the concentration of dihydrochalcones was comparable between WF and RF genotypes (Fig. S5), it is unlikely that *CHS* and the upstream structural genes in the pathway may play a role in the different kaempferol 3-*O*-glycosides and anthocyanin concentrations between WF and RF genotypes. Compared with quercetin, kaempferol lacks one more hydroxyl group at the 3′-position of the B-ring, so the glycosylation at the 3-position of the C-ring of kaempferol and quercetin might be catalyzed by the same UGT gene. At different developmental stages, the concentrations of quercetin 3-*O*-glycosides were comparable or even lower in the flowers of WF compared with that of RF genotypes (Fig. 3, Fig. S4). So, the difference in the concentrations of kaempferol 3-*O*-glycosides between WF and RF should not necessarily relate to glycosylation.

In previous studies, it was found that the overexpression of both *MdF3*′*HI* and *MdF3*′*HII* in either the *Arabidopsis tt7* mutant or tobacco would recover the seed pigmentation of the *tt7* mutant or result in more anthocyanin accumulation in tobacco flowers^3^. Tian et al. also reported that the ever-red leaf colouration of crabapple was regulated by *MYB10* TF through regulating the expression level of *F3*′*H*^33^. However, based on the gene expression levels in the different *Malus* genotypes as well as that in the *MYB10*-overexpressed ‘Royal Gala’ transgenic lines, the competition between anthocyanin and kaempferol 3-*O*-glycosides synthesis in *Malus* flowers might be controlled by the genes such as *F3*′*HII*, *FLS, DFR*, and *ANS* (Fig. 4, Fig. S7). For anthocyanin and kaempferol 3-*O*-glycosides synthesis, *F3*′*H* and *FLS* are the key enzymes controlling the metabolic flux using the shared substrates such as naringenin or dihydrokaempferol (Fig. S6). So, compared with *DFR* and *ANS*, *F3*′*HII* and *FLS* might contribute more to the competition. *MdF3*′*HI* has 91% nucleotide sequence identity in the coding region to *MdF3*′*HII* (Fig. S11), but unlike *F3*′*HII*, the expression of *F3*′*HI* in flowers was not significantly different between WF and RF genotypes (Fig. 4). The overexpression of *MYB10* hardly changes the expression of *F3*′*HI* compared with that of *F3*′*HII* in the red flowers of ‘Royal Gala’ either (Fig. S8).

Interestingly, it was found that kaempferol 3-*O*-glycosides accumulated mainly in flowers but not in leaves or fruits of *Malus* (Fig. 3). The function of compounds usually relates to the tissues/organs they are accumulated in. In the leaves, whilst expression of *F3*′*HII* was downregulated sharply, the level of kaempferol 3-*O*-glycosides only increased a small amount (Fig. 5d–f). This suggests kaempferol 3-*O*-glycosides should be functional mainly in flowers. This might also explain why the concentrations of quercetin 3-*O*-glycosides remained unchanged in the transgenic leaves (Fig. 5f). Recently, it was reported that flavonols in the pollen of tomato might control pollen tube growth and integrity by regulating ROS homeostasis during high-temperature stress^23^. Meng et al. reported that decreased sorbitol synthesis might lead to abnormal stamen development and reduced pollen tube growth^34^. In the present study, the difference in kaempferol 3-*O*-glycosides concentration between WF and RF did not affect pollen viability (Fig. S9). Moreover, no kaempferol 3-*O*-glycosides was found in the pollen of *Malus* (Fig. S2). This suggests that before pollination, the development of pollen was not affected by kaempferol 3-*O*-glycosides or sorbitol in the coat of pollen sacs. After pollination, the reduced kaempferol 3-*O*-glycosides concentration associated with *MYB10*-overexpression in ‘Royal Gala’ appeared to slow pollen tube growth (Fig. 6), demonstrating the regulation of pollen tube growth by kaempferol 3-*O*-glycosides in pistils. The effects of various flavonoid compounds on pollen tube growth in vitro also clearly showed that it was kaempferol 3-*O*-glycosides, especially kaempferol 3-*O*-rhamnoside rather than cyanidin 3-*O*-glycosides or quercetin 3-*O*-glycosides, that regulated pollen tube growth of *Malus* (Fig. 7).

Pollen tubes interact extensively with pistils during growth. Efficient tip growth requires structural organization and vesicular trafficking activities to be highly coordinated in pollen tubes, implying a requirement for signaling networks to coordinate these subcellular behaviors during pollen-pistil interactions. For instance, the signaling pathways involving ROP GTPases, calcium, and phosphoinositides have been demonstrated to coordinate pollen tube growth by regulating cellular activities, such as actin dynamics, exocytosis, and endocytosis^35^. Recently, it was found that the pollen tube tip-localized Pollen receptor-like kinase 6 (PRK6) regulated the direction of pollen tube tip growth as an essential receptor for AtLURE1 signaling^36^, while the ADAPTOR PROTEIN-3 (AP-3) complex-mediated pollen tube growth in Arabidopsis by coordinating vacuolar targeting and organization^37^. In the present study, it was found that kaempferol 3-*O*-rhamnoside might regulate the pollen tube growth through the ROP GTPases, calcium and phosphoinositide signaling pathway, supported by gene expression data and calcium imaging results (Figs. 9, 10). Moreover, the present study also indicates that kaempferol 3-*O*-rhamnoside regulated these signaling pathways through modifying auxin transport (Fig. 10).

Auxin has been shown to be critical for pollen development. External applications of auxin can stimulate in vitro pollen tube growth of *Nicotiana tabacum* and *Torenia fournieri*^38,39^. Studies on the pollen-specific auxin efflux carrier PIN8 also support a role for auxin in pollen tube growth^40,41^. HSPs were suggested to be involved in the thermal tolerance of pollen tubes at high temperatures^42^. Interestingly, in our studies, it was observed that some HSPs were involved in the auxin signaling pathway during the regulation of pollen tube growth (Figs. 8–10). With the inhibition of auxin transport, the expression levels of *HSPs* were downregulated, while the levels were either unchanged or enhanced when calcium signaling was blocked. These results suggest that HSPs were downstream of auxin signaling but upstream of the calcium signaling pathway. It has been reported that there are over 100 receptor-like kinases expressed in pollen tubes, most of which are expected to be the receptors of small peptides^43^. Functioning as low-molecular-weight signaling peptides, HSPs-receptor signaling modules appear to be predominant in pollen tube growth. We would not expect reactive oxygen species signaling to be involved in the regulation of pollen tube growth, as kaempferol 3-*O*-glycosides has only one hydroxyl group while quercetin 3-*O*-glycosides and cyanidin 3-*O*-galactoside have an *o*-dihydroxyl at the B-ring. The *o*-dihydroxyl at the B-ring means relatively higher antioxidant capacity^25,26^, and the determinacy to react with the reactive oxygen species.

Pollen tube growth is the main factor which influences seed set^44,45^. Flavonols could regulate seed set in tomato through affecting pollen tube growth^23^. So, it is possible that the exogenous application of kaempferol 3-*O*-rhamnoside could increase the amount of well-developed seeds in the RF genotypes of *Malus* (Fig. 8), and account for transgenic ‘Royal Gala’ red fruit having less well-developed seeds (Fig. 1). For RF genotypes, less kaempferol 3-*O*-glycosides resulted in less well-developed seeds, which would mean lower reproductive capacity in natural conditions. Other studies report that bees seem to be the most important pollinator for apple, and prefer yellow or white flowers with UV absorption properties^46–49^. Therefore, the WF genotypes might also have an advantage in attracting bees for pollination compared with the RF genotypes of *Malus*. Taken together, this may go some way to explaining why surviving wild *Malus* species have predominantly white flowers.

**Fig. 11:**
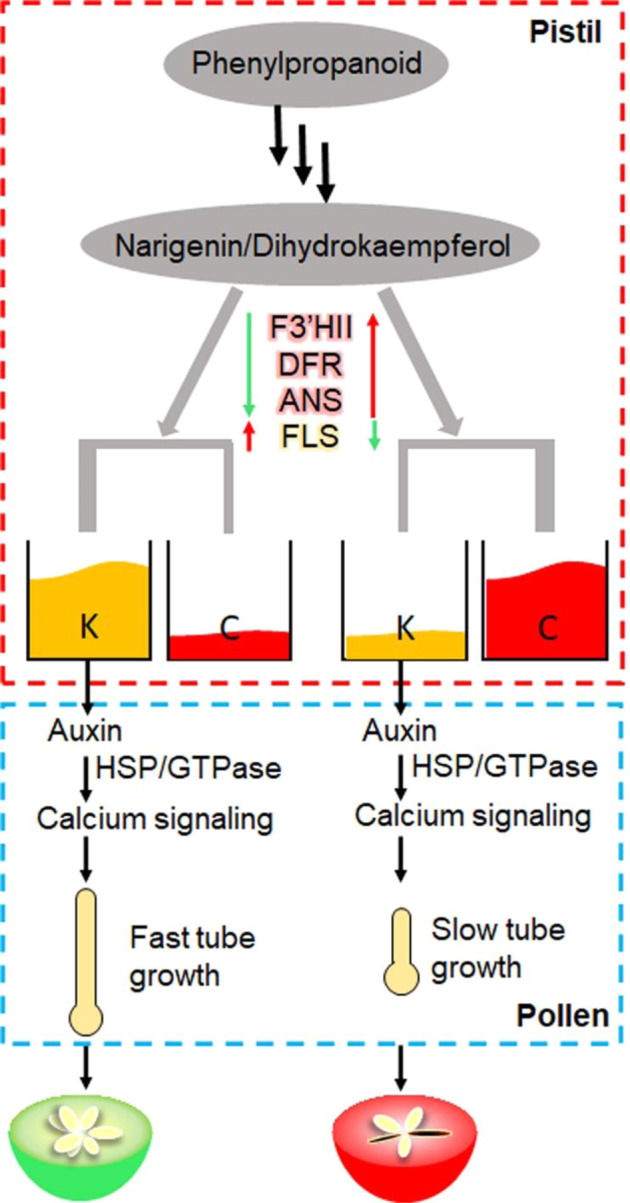
The model of effects of the competition between cyanidin 3-*O*-glycosides and kaempferol 3-*O*-glycosides biosynthesis in flowers on the pollen tube growth and seed set of *Malus.* F3′HII, flavonoid 3′-hydroxylase II; DFR, dihydroflavonol 4-reductase; ANS, anthocyanin synthase; FLS, flavonol synthase; K, kaempferol 3-*O*-glycosides; C, cyanidin 3-*O*-glycosides; HSP, heat shock protein

In summary, we showed that *F3*′*HII*, *FLS, DFR*, and *ANS* might control the competition between kaempferol glycosides and anthocyanin biosynthesis in *Malus* flowers (Fig. 11). Kaempferol glycosides presence in pistils coordinated auxin transport, the gene expression of HSPs and ROP GTPases, and calcium signaling in pollen tubes, affecting pollen tube growth and seed set of *Malus*.

## Materials and methods

### Plant materials and isolation of compounds

Fourteen *Malus* genotypes including four apple cultivars (*M. domestica* ‘Golden Delicious’, *M. domestica* ‘Fuji’, *M. domestica* ‘Red Delicious’, *M. domestica* ‘Gala’), four wild species (*M. hupehensis*, *M. micromalus*, *M. halliana*, *M. baccata*), and six ornamental crabapple cultivars (*M*. ‘Sparkler’, *M*. ‘Radiant’, *M*. ‘Adams’, *M*. ‘Kelsey’, *M*. ‘Perfect Purple’, *M*. ‘Royalty’) were used in this study. They were grown in the horticultural experimental orchard of Northwest A&F University, Yangling, Shaanxi, China. All trees were grown under standard horticultural practices with disease and pest control. Flower petals were collected at different developmental stages (stage 1, 6 days before full bloom; stage 2, 3 days before full bloom; and stage 3, full bloom). At stage 3, the four apple cultivars and four wild species had white flowers while at stages 1 and 2, the flowers were pink. The six ornamental crabapple cultivars always had red flowers. Pistils, stamens, and pollens were collected 1 day before full bloom. New fully expanded leaves were collected at the full bloom stage, while fruit were collected at ~40 days after full bloom. Five replicates (one tree for each replicate. For each tree, 20 flowers, fruits, or leaves were taken from the west part of tree canopies at each sampling time) of all tissues were collected and immediately frozen in liquid nitrogen, then ground to powder and mixed in liquid nitrogen with an Al l grinder from IKA^®^ works (VWR, Radnor, PA, USA), and stored at −80 °C until use. Pollen were dried at 28 °C for 24 h and stored at −20 °C until use. The *MdMYB10* overexpressed ‘Royal Gala’ apple lines and the corresponding WT trees were grown in glasshouse conditions, as described by Espley et al.^31^.

For compound isolation, 5 kg of *M. domestica* ‘Fuji’ flowers and 0.8 kg of *M*. ‘Perfect Purple’ leaves were collected. The dried flowers were used to isolate kaempferol derivative compounds and quercetin derivative compounds (quercetin 3-*O*-galactoside and quercetin 3-*O*-rhamnoside) whereas the leaves were used for cyanidin 3-*O*-galactoside isolation, as described by Xiao et al.^25^ with minor modifications. The isolated kaempferol derivative compounds (purity > 98%) were identified using LC-ESI-MS and NMR as described by Xiao et al.^25^.

### Transformation of apple

A line with high regeneration capacity isolated from ‘Royal Gala’ (*M. domestica*) named ‘GL-3’^50^ was used for genetic transformation with *MdF3*′*HIIa*. Transgenic ‘GL-3’ apple plants were generated from leaf fragments through *Agrobacterium*-mediated transformation, as previously described^50^. The vector pK7GWIWG2 driven by the 35S promoter was used as the RNA interference vector for *MdF3*′*HIIa*. The *MdMYB10* overexpressed ‘Royal Gala’ apple plants and the corresponding WT were as described by Espley et al.^31^.

### Flavonoid compound analysis

The extraction and analysis of flavonoid compounds in *Malus* were carried out as described by Li et al.^51^. The isolated compounds with the purity over 98% with different concentrations were used to make standard curves to calculate the concentrations of each compound in plants.

### Seed set analysis

Fruits of *Malus* were harvested 60 days after full bloom, with 60 fruits from 5 trees being randomly selected, seeds extracted and a knife cut to the side of the seed used to reveal the ventricle. For the transgenic fruit and its wild type, 15 fruit were, respectively, selected randomly for seed set analysis. The image of seed sets was observed using a stereoscopic microscope (Leica MZ10F, Germany).

### Flavonoid compounds treatment and phenotypic analysis of pollen tube growth in vitro

Harvested pollen grains were cultured on solid germination medium (GM, 10% sucrose, 0.01% boric acid, 1% agar, pH 6.5) as described by Meng et al. with minor modification^34^. For compound treatment, 1 μM kaempferol, kaempferol derivatives, quercetin derivatives, or cyanidin 3-*O*-galactoside dissolved in Dimethylsulfoxide (DMSO) were added into the GM and mixed uniformly before the media solidified. The DMSO solution without additives was used as the control. The pollen was incubated at 28 °C in a dark and humid environment for 4 h.

The pollen of *M*. *domestica* ‘Fuji’ was used for 1-*N*-naphthylphthalamic acid (NPA, final concentration, 40 μM), β-naphthoxyacetic acid (NOA, final concentration 20 μM), and EGTA (final concentration 10 mM) treatments. In our preliminary experiment, it was found that EGTA fully inhibited the germination of pollen, so the EGTA treatments were carried out after 2 h of culture of pollen.

The calcium fluorescence and pollen tube length were observed with a fluorescence microscope (BX51 + DP70, Olympus). The length of pollen tube was measured by ImageJ. Measurement of the length of pollen tubes was carried out on five replicates. For each replicate, 50 pollen grains were randomly selected.

### Phenotypic analysis of pollen tube growth in vivo

Pollen grains from *M. domestica* ‘Granny Smith’ were collected to pollinate ‘Royal Gala’ and its *MYB10* overexpressed transgenic flowers. The pistils were harvested 24, 48, and 72 h after pollination, and then fixed in FAA fixative solution (70% alcohol: acetic acid: formaldehyde = 18:1:1) for 12 h. The fixed pistils were washed with distilled water three times following an incubation in 2 M NaOH softening solution for 24 h. After being washed with distilled water three times, the pistils were stained in 0.1% aniline blue (0.1% K_3_PO_4_ buffer) for 24 h in the dark. Pollen tubes in the style were visualized with a BX51 + DP70 fluorescence microscope (Olympus Corporation, Japan) as described by Kho and Baer^53^ with minor modifications.

### RNA extraction, qRT-PCR, and RNA-seq analyses

Total RNA was isolated using the SDS-phenol method according to Malnoy et al.^52^. All qRT-PCR experiments were performed with the Bio-Rad CFX96 system (Bio-Rad Laboratories, Hercules, CA, USA). The primers for *MdActin*, *MdF3*′*HI*, *MdF3*′*HII*, *MdFLS*, *MdDFR*, and *MdANS* were shown in Supplemental Table S3. The total RNA was also submitted to Novogene in Beijing (https://en.novogene.com/) for library construction and RNA sequencing. Unigenes with adjusted *P*-value <0.05 and fold change Log_2_^x^ > 0.2 were identified as DEGs. Three biological replicates were performed for the analysis.

### Chemical feeding to stigma in vivo

One day before full bloom, 50 or 100 μM kaempferol 3-*O*-rhamnoside was dropped onto the stigma of two red flower genotypes, *M*. ‘Adams’ and *M*. ‘Radiant’, while 50 or 100 μM cyanidin 3-*O*-galactoside was dropped to the stigma of two white flower genotypes, *M. micromalus* and *M. halliana*. The flowers treated with the DMSO solution without additives were used as a control. Forty days after feeding treatment, the seed set was observed and counted. For each treatment, 60 flowers from five trees were randomly selected for feeding treatment. The fruit setting rate was 100%.

### Statistical analysis

All data were analyzed with *t*-test or least significant difference (LSD) using SPSS 16.0 software (SPSS, Chicago, IL, USA).

## Supplementary information

Supplementary Figures

Supplementary tables
