# Supplementary material for: Competition between anthocyanin and kaempferol glycosides biosynthesis affects pollen tube growth and seed set of Malus
Source: Hortic Res. 2021 Aug 1;8:173. doi: 10.1038/s41438-021-00609-9 (PMC8325685; doi:10.1038/s41438-021-00609-9)
Supplement: Supplementary file 1 — Supplementary Figures [file 41438_2021_609_MOESM1_ESM.docx]

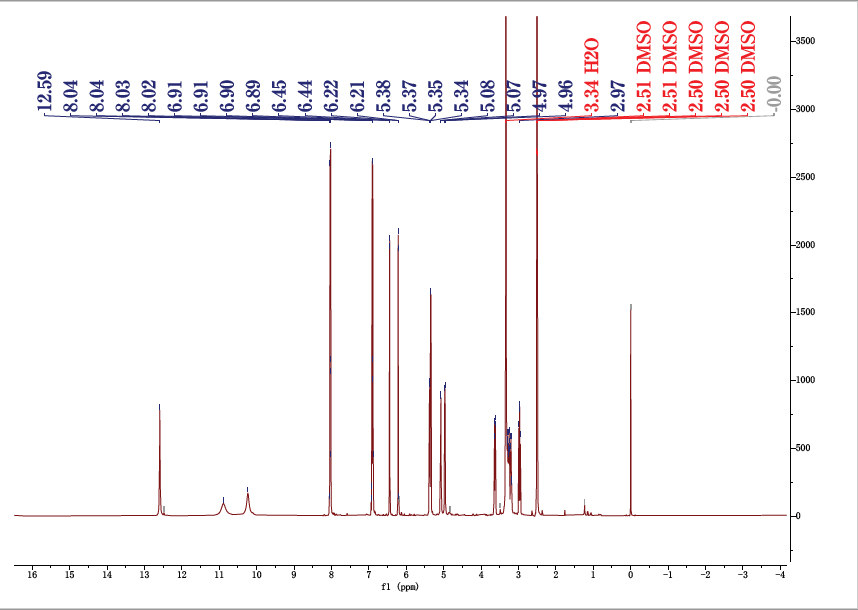


^1^H-NMR map of K1


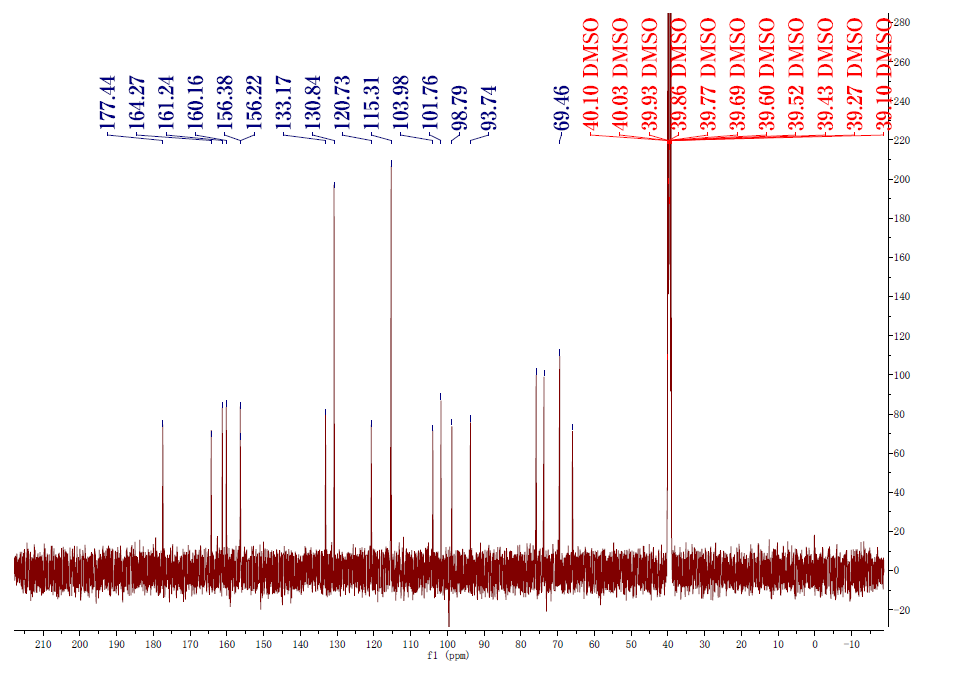


^13^C-NMR map of K1


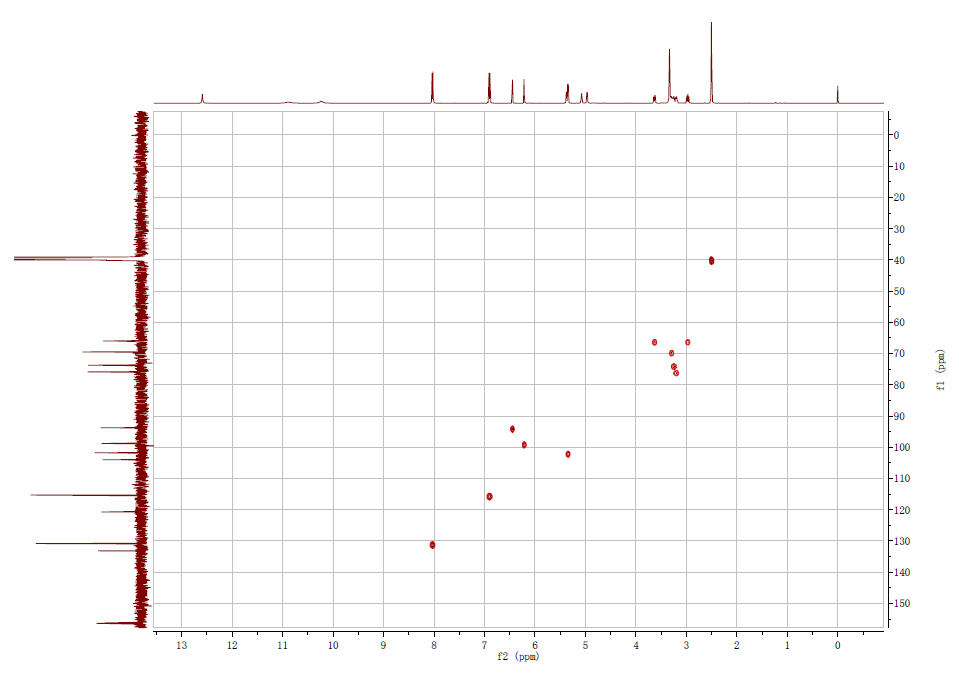


HSQC map of K1


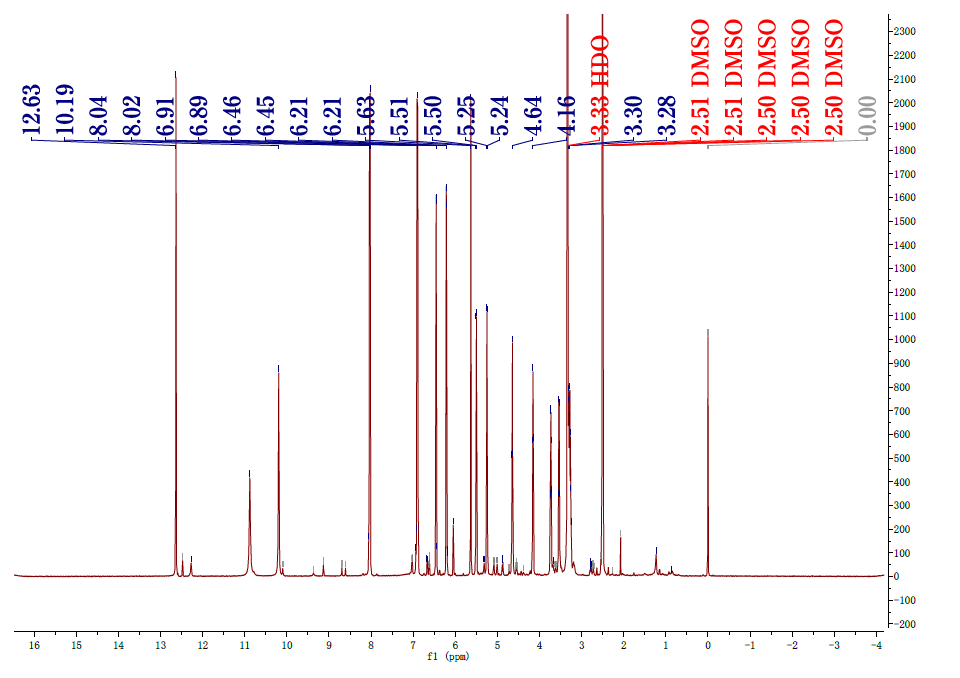


^1^H-NMR map of K2.


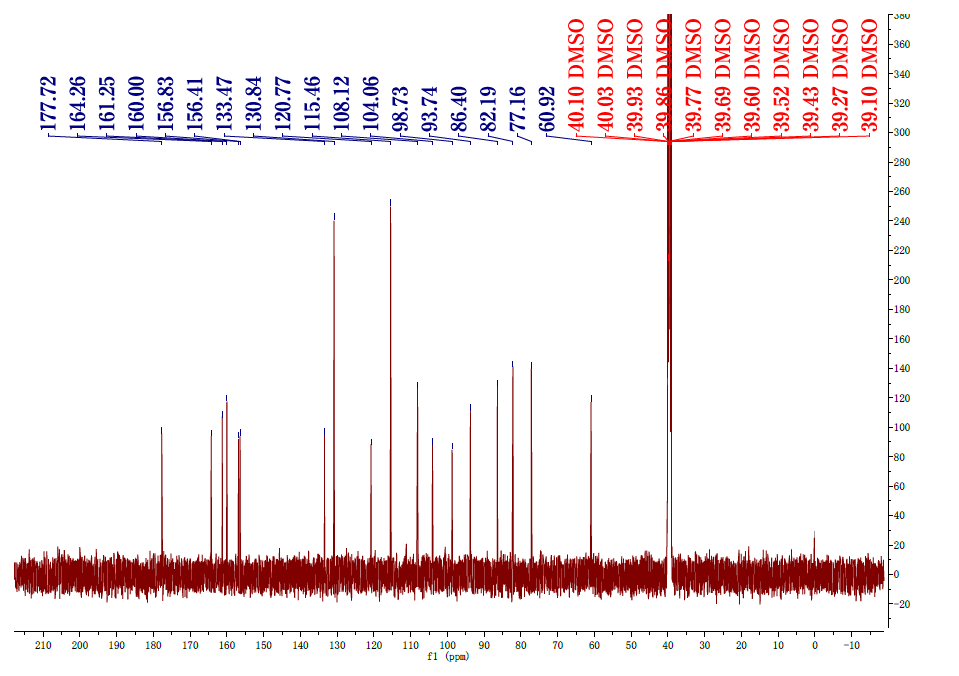


^13^C-NMR map of K2.


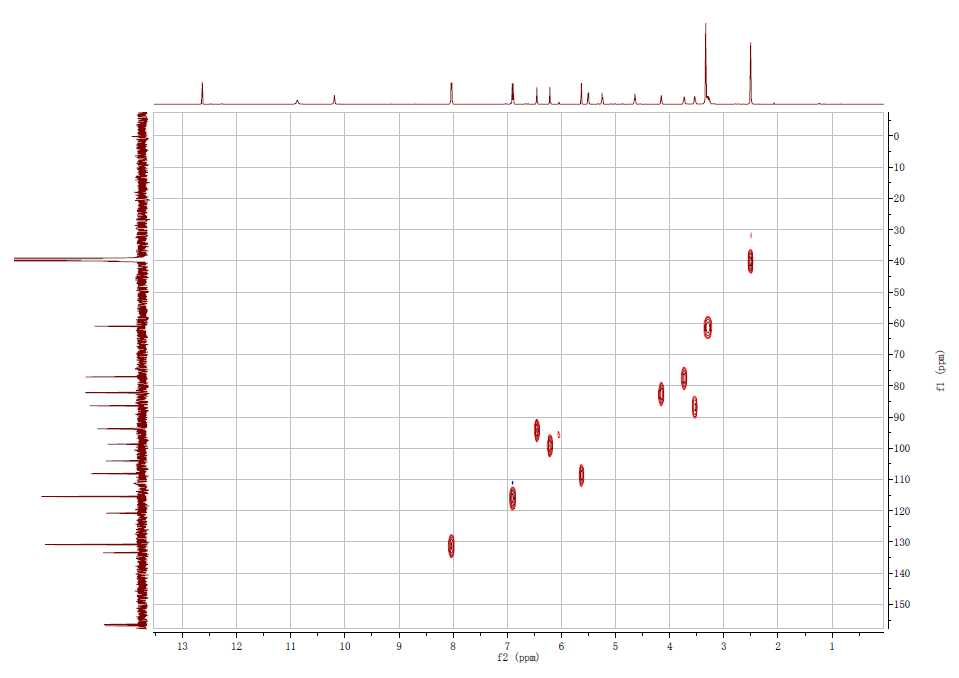


HSQC map of K2.


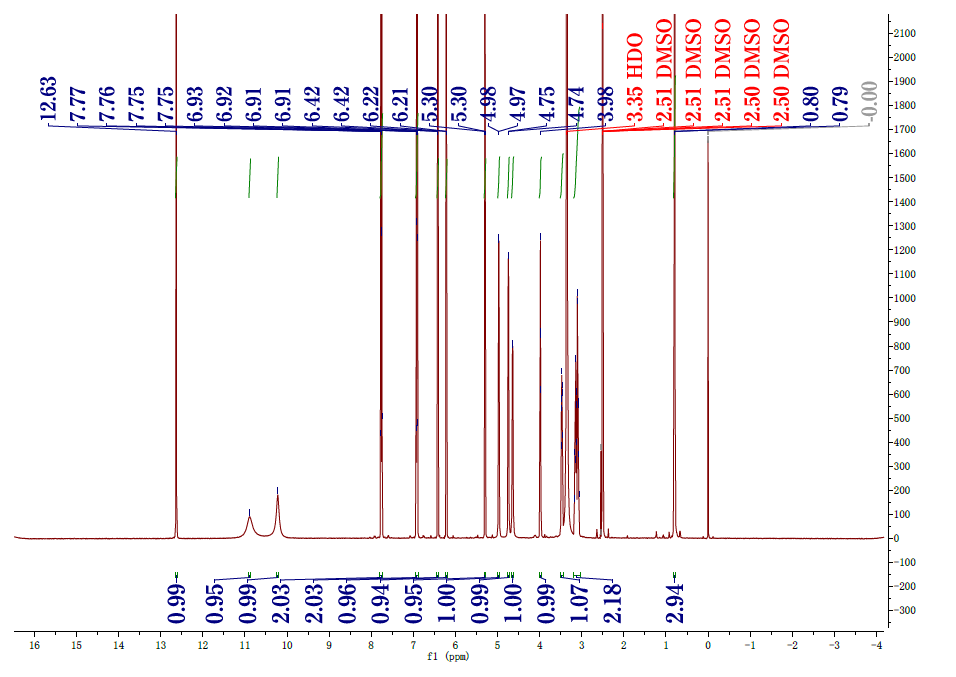


^1^H-NMR map of K3.


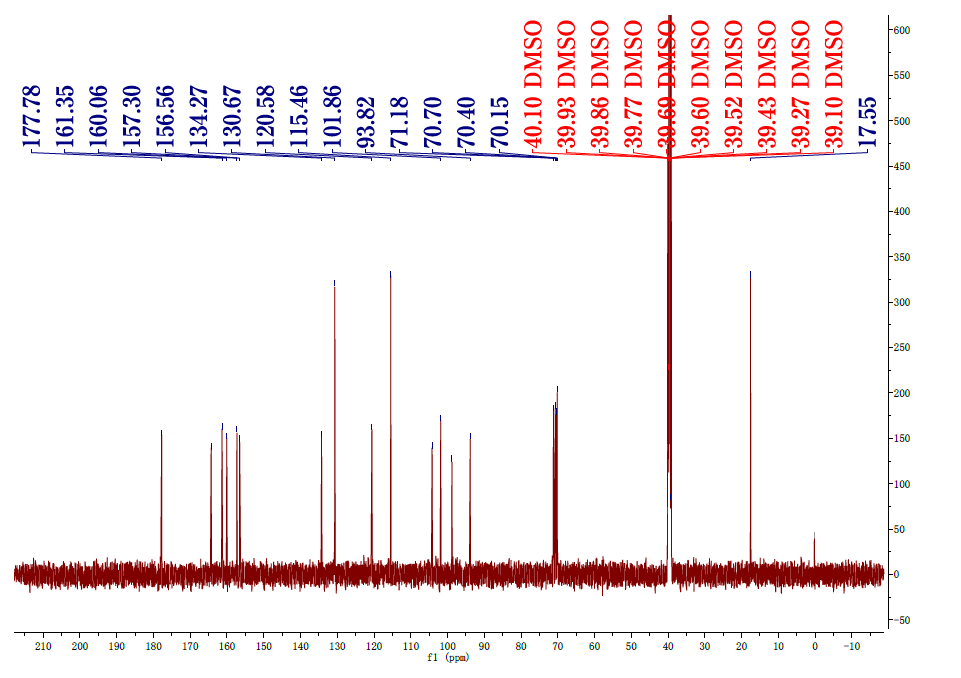


^13^C-NMR map of K3.


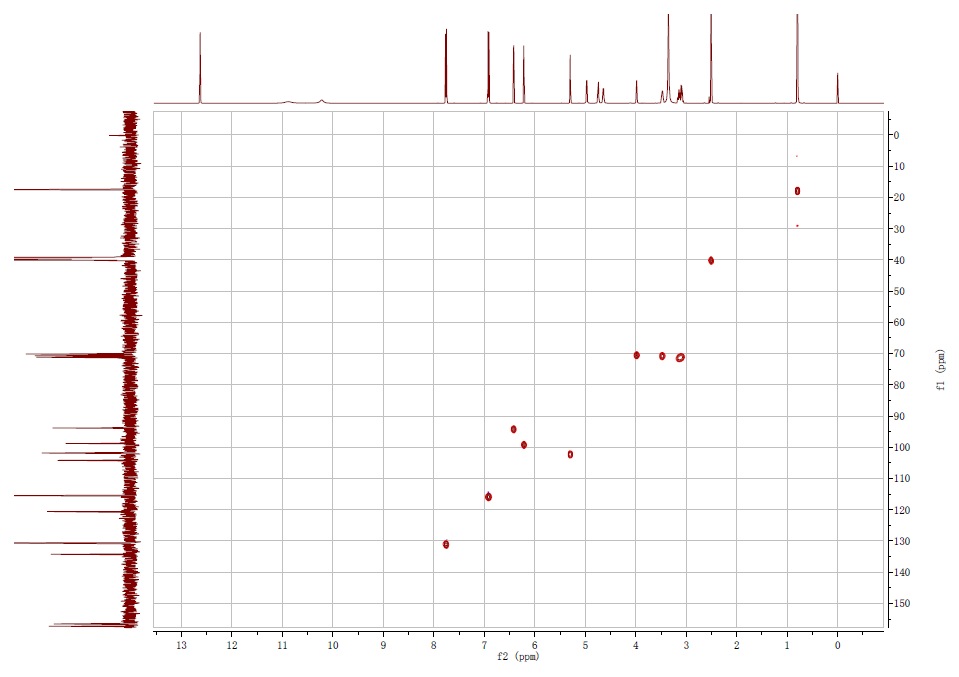


HSQC map of K3.

**Fig. S1 The NMR spectra of kaempferol 3-*O*-glycosides.**

K1, kaempferol 3-*O*-xyloside; K2, kaempferol 3-*O*-arabinoside, and K3, kaempferol 3-*O*-rhamnoside.


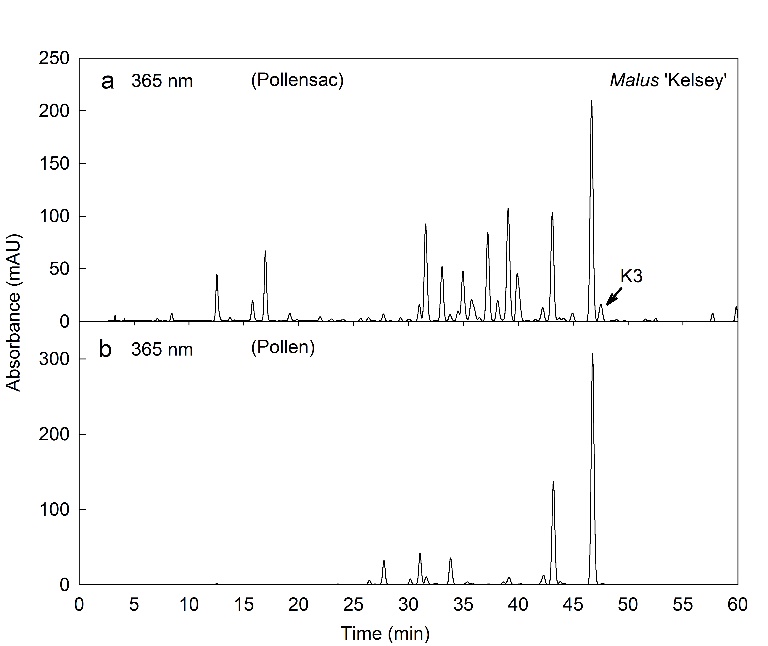

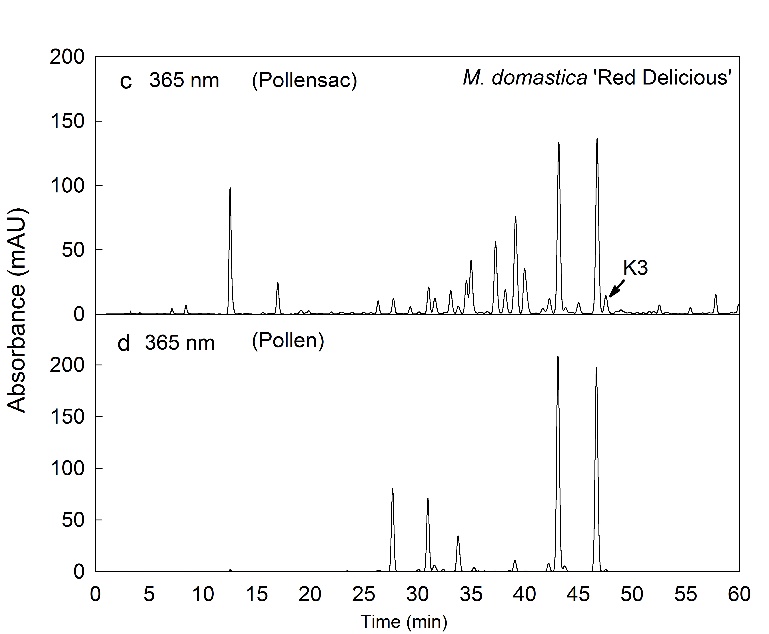

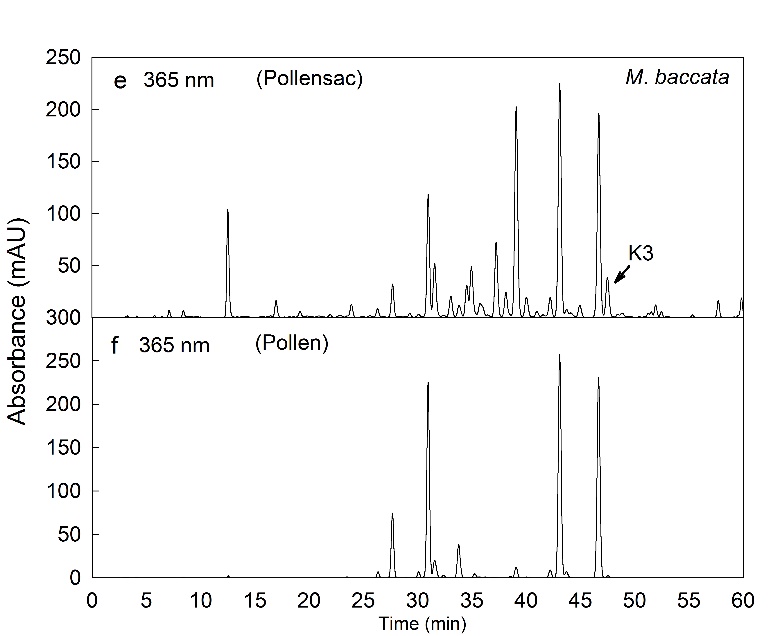


**Fig. S2 The chromatogram of flavonoids in pollensacs and pollens of *Malus*.**

**K3, kaempferol 3-*O*-rhamnoside.**


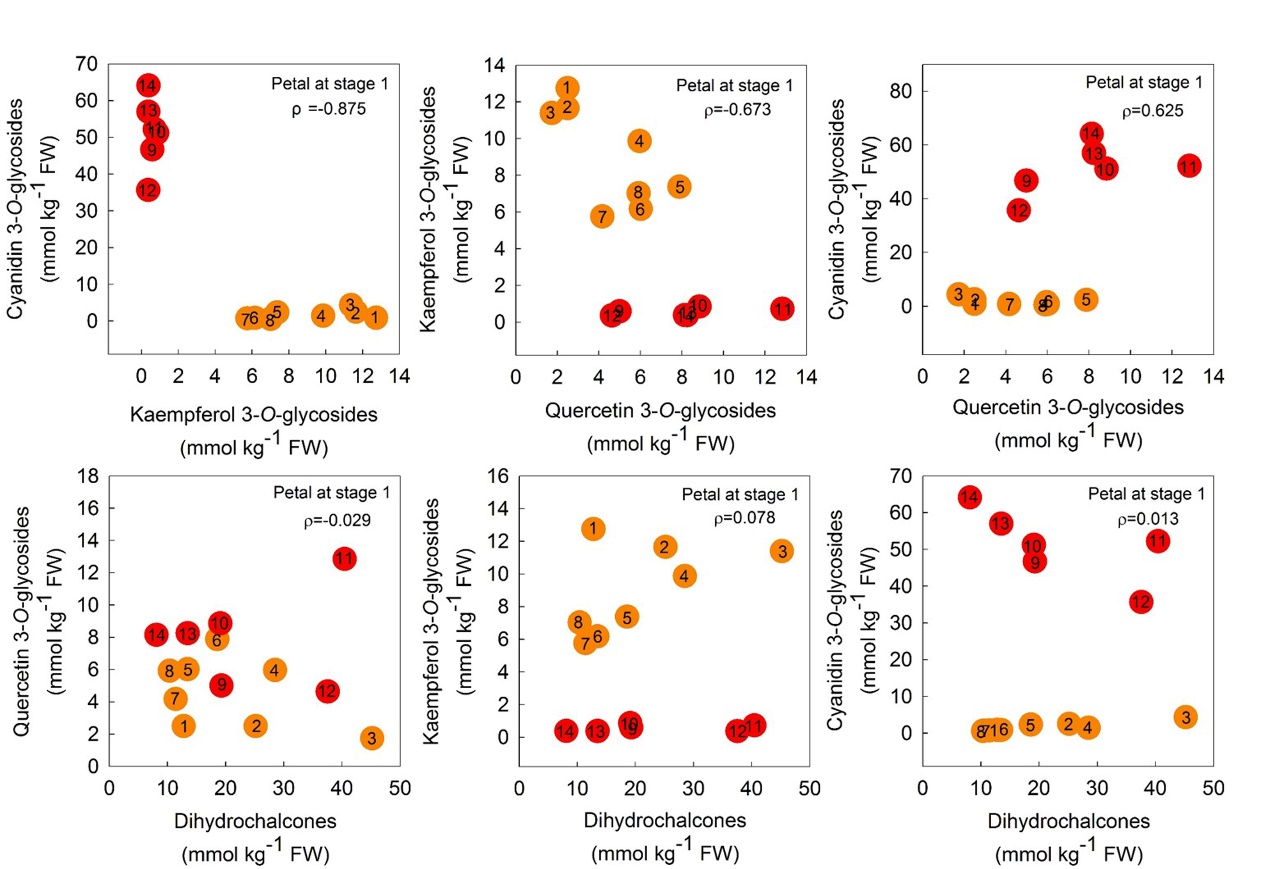


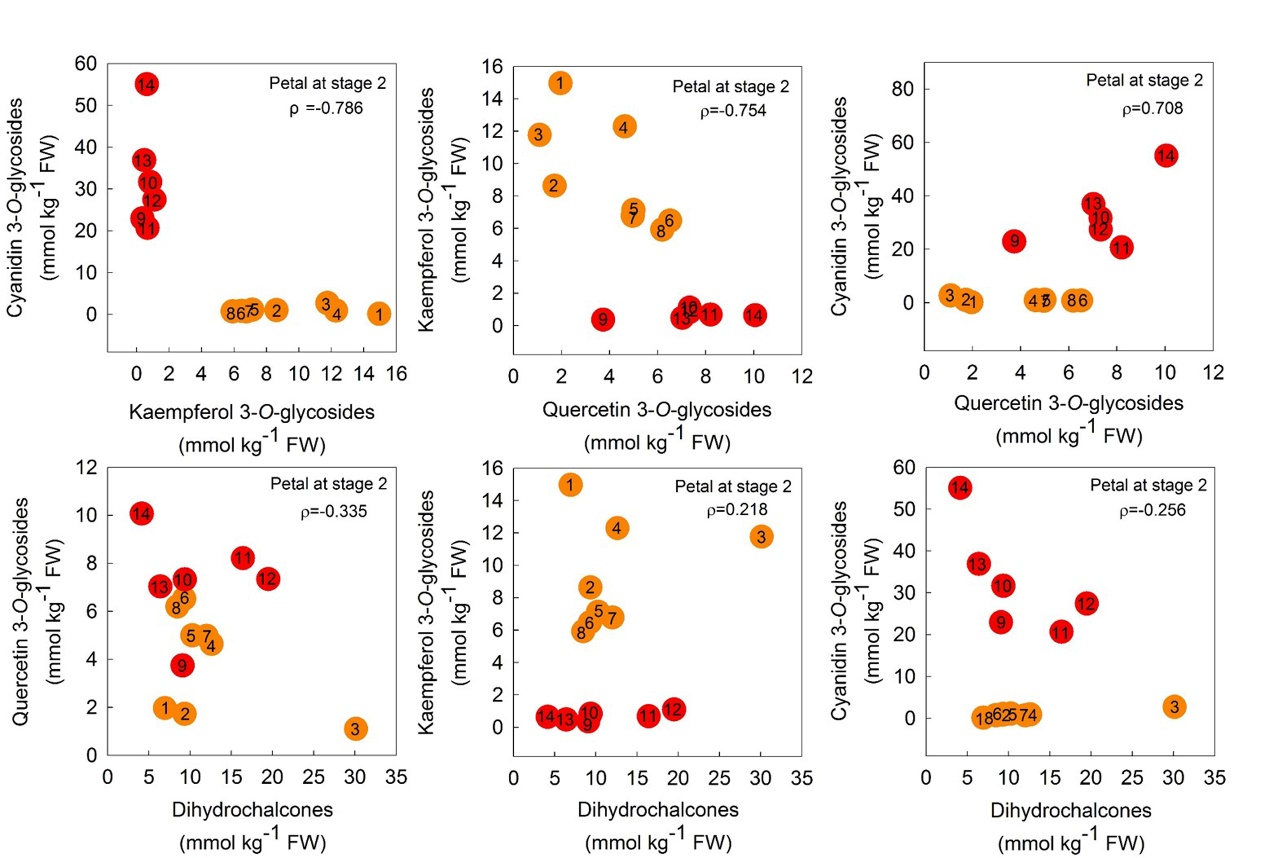


Continue


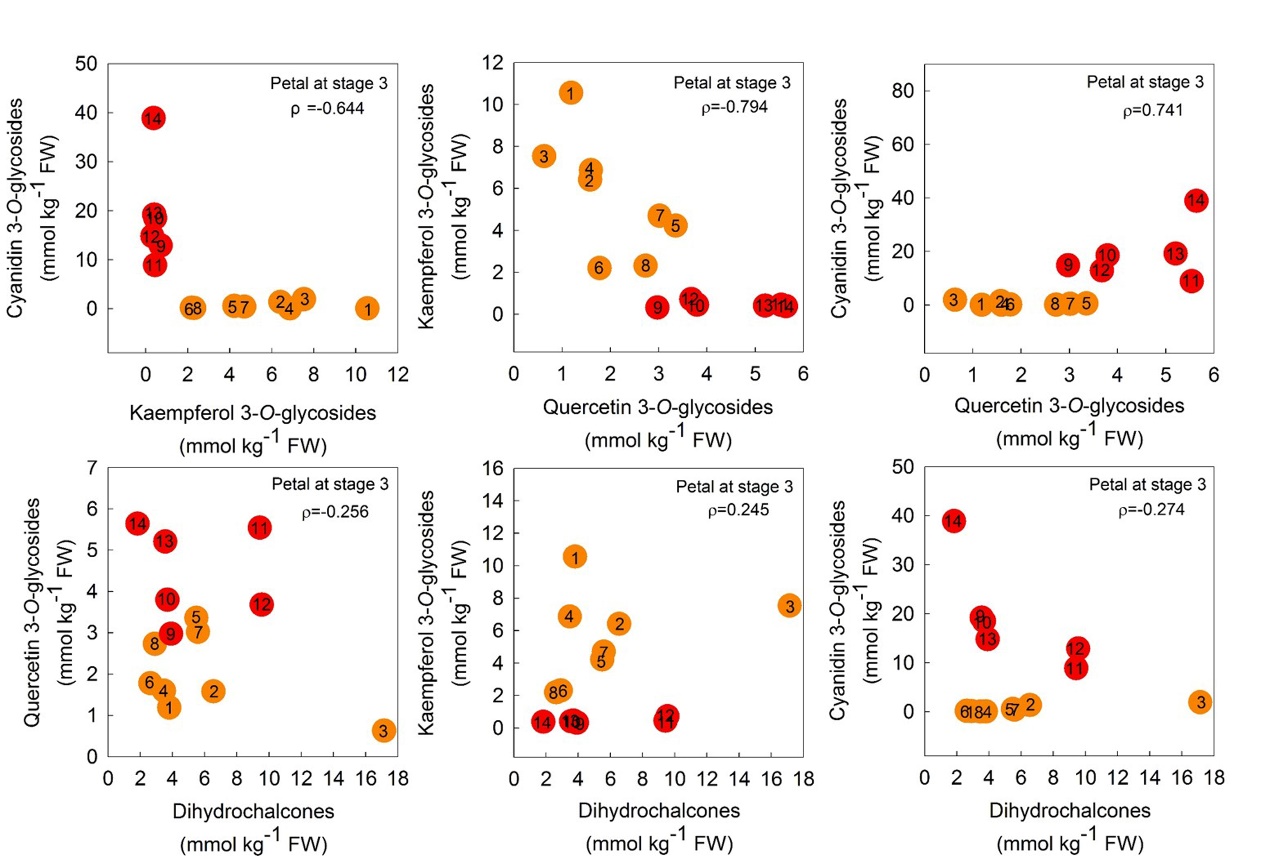


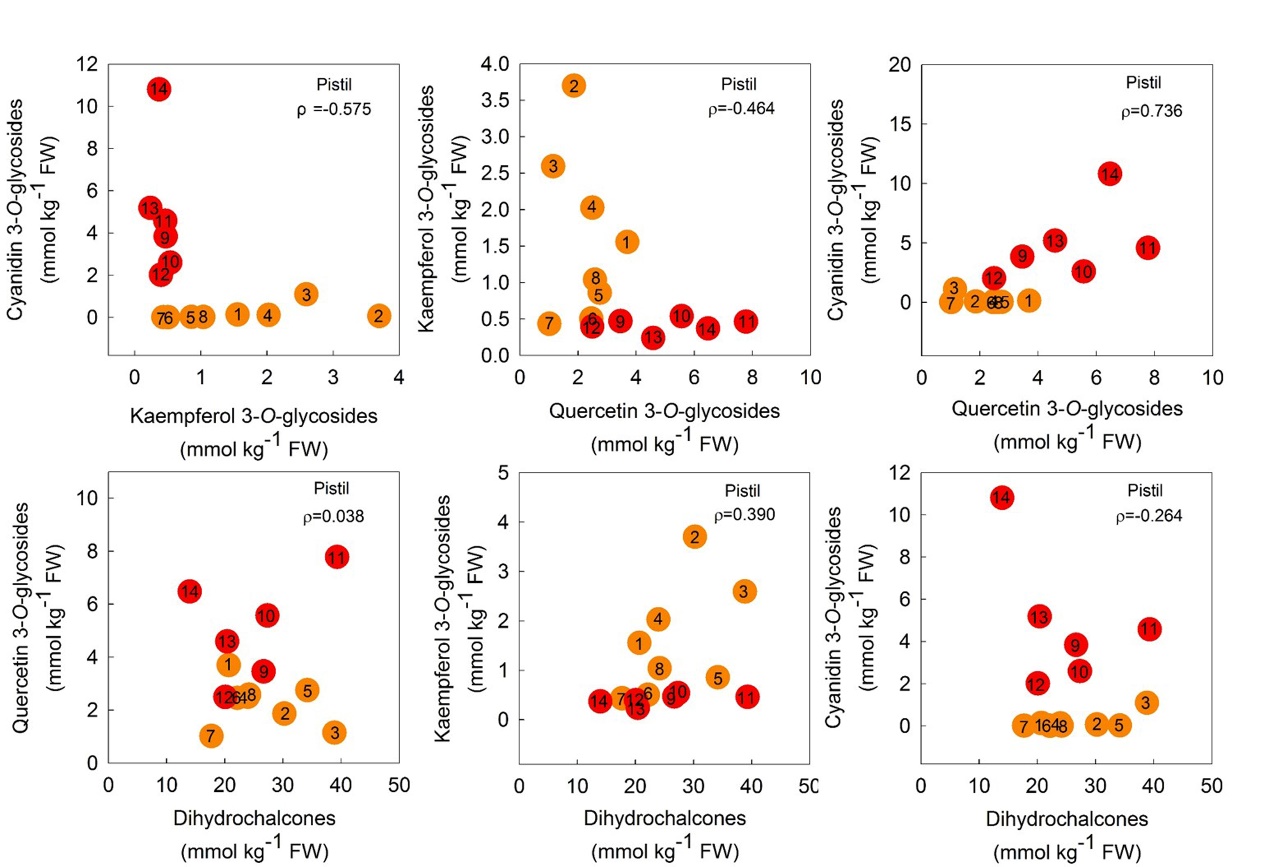


Continue


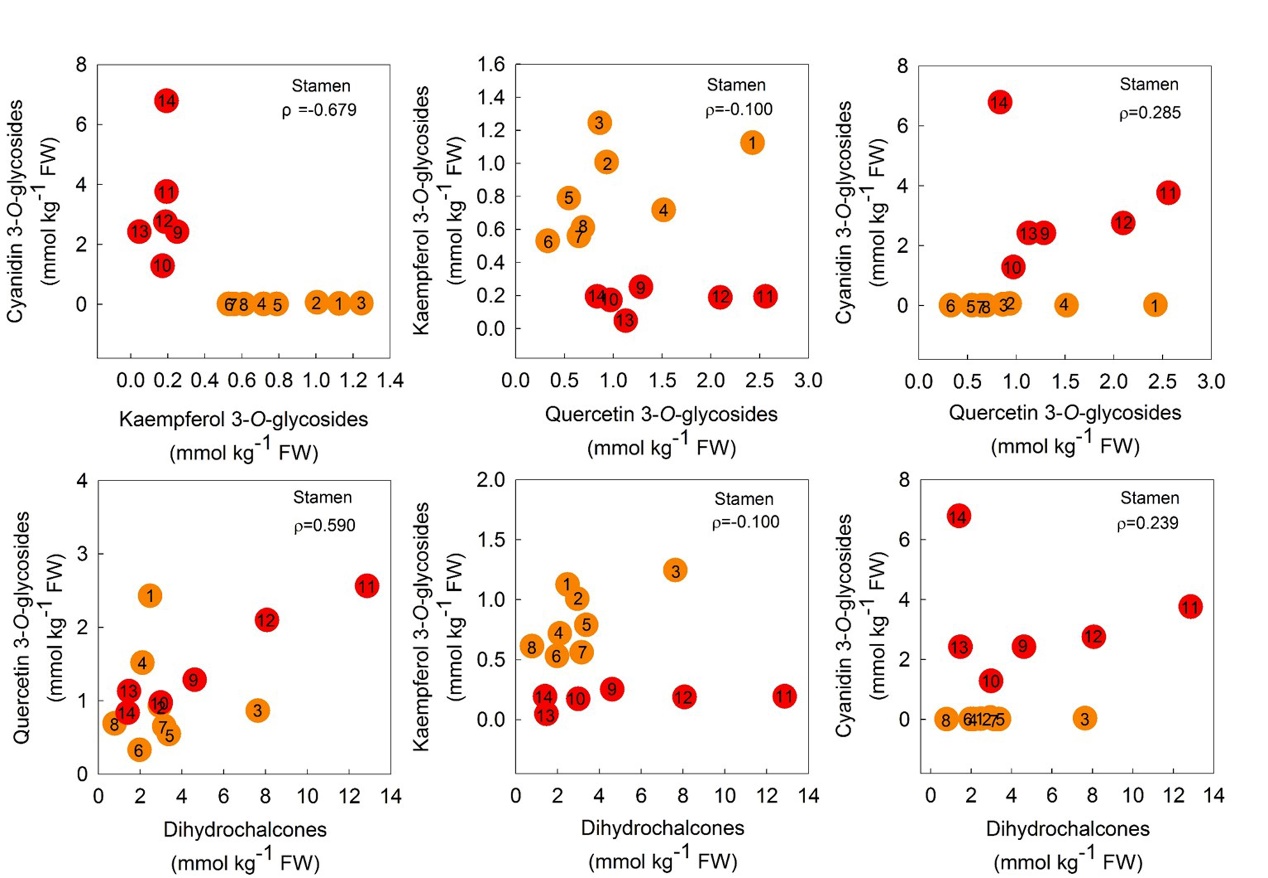


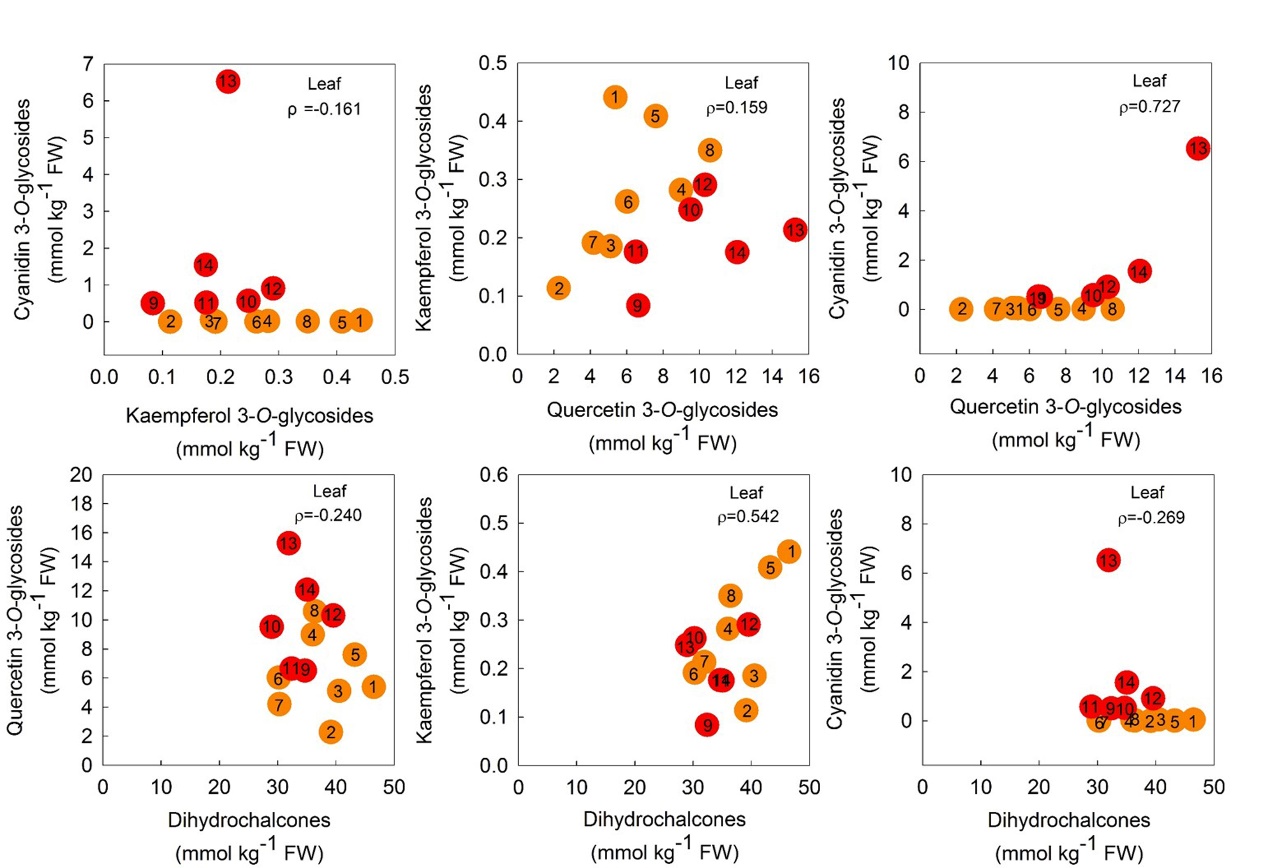


Continue


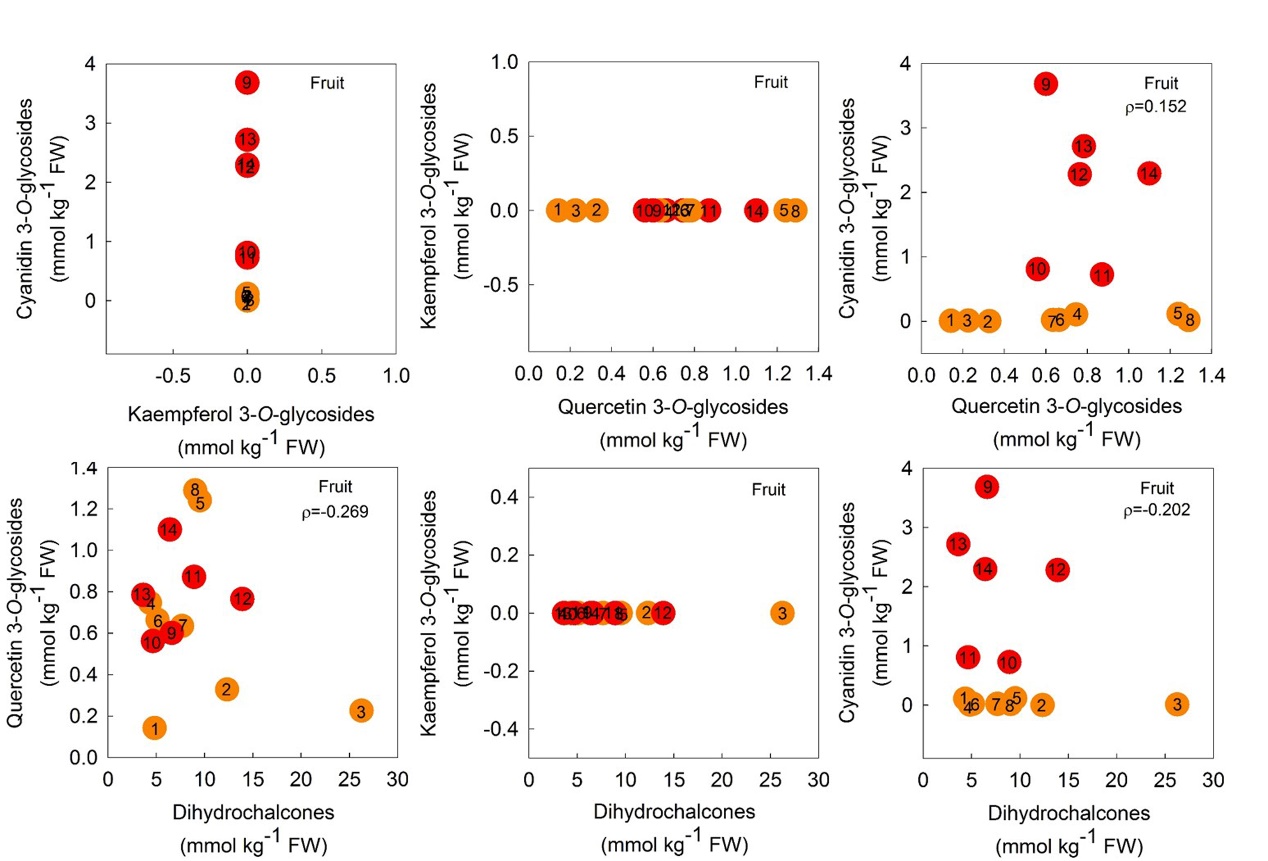


**Fig. S3 Correlations between different flavonoid compounds in different tissues of *Malus*.**


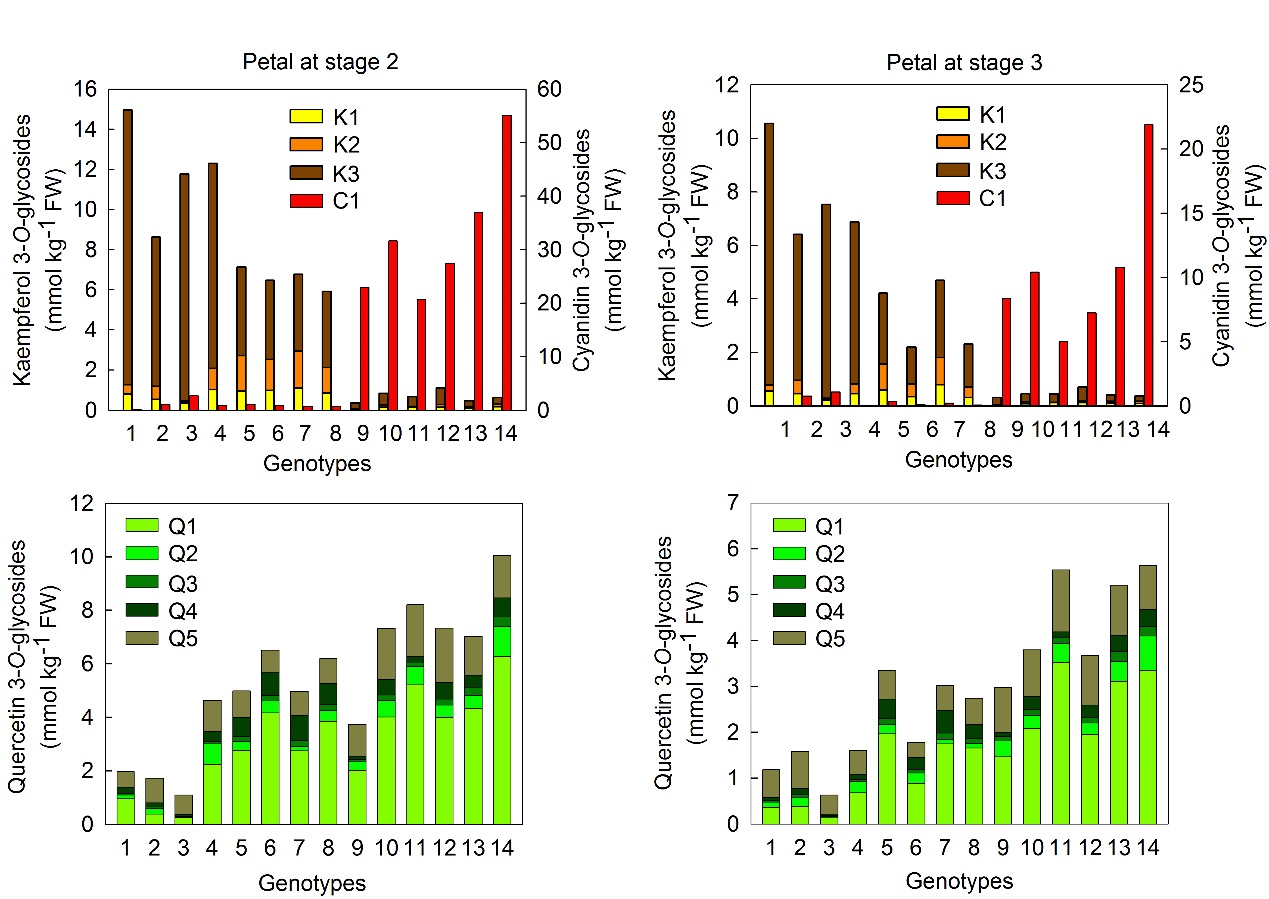


**Fig. S4 Flavonoid compounds concentration in flower petals of *Malus* germplasm resources at different stages.**

1, *M. hupehensis*; 2, *M. micromalus*; 3, *M. halliana*; 4, *M. baccata*; 5, *M. domestica* ‘Golden Delicious’; 6, *M. domestica* ‘Fuji’; 7, *M. domestica* ‘Red Delicious’; 8, *M. domestica* ‘Gala’; and red flower genotypes: 9*, M.* ‘Sparkler’; 10, *M.* ‘Radiant’; 11, *M.* ‘Adams’; 12, *M.* ‘Kelsey’; 13, *M.* ‘Perfect Purple’; 14, *M.* ‘Royalty’. C1: cyanidin 3-*O*-galactoside; K1, kaempferol 3-*O*-xyloside; K2, kaempferol 3-*O*-arabinofuranoside, K3, kaempferol 3-*O*-rhamnoside.


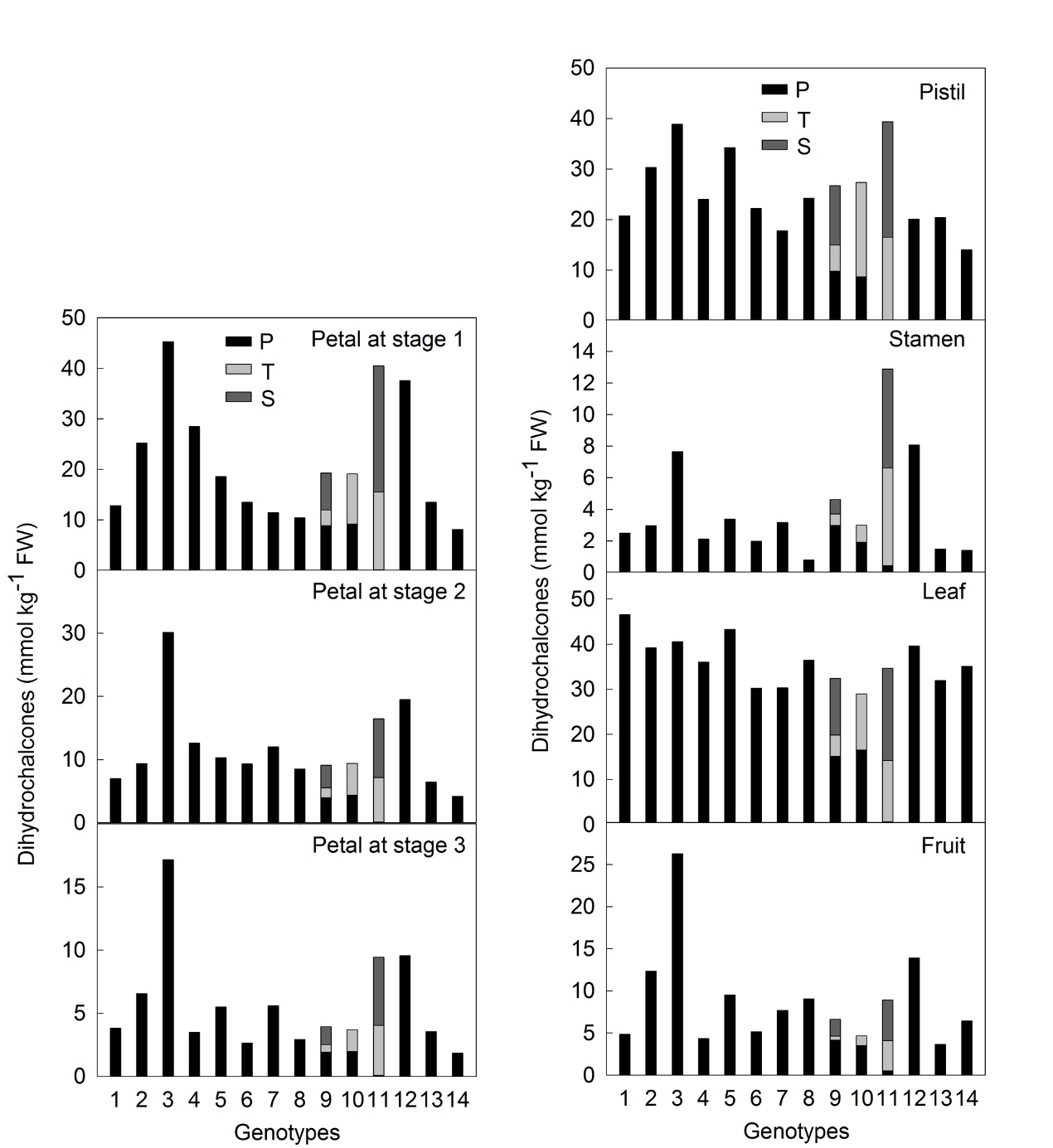


**Fig. S5 Dihydrochalcone concentrations in different tissues of *Malus* germplasm resources.**

1, *M. hupehensis*; 2, *M. micromalus*; 3, *M. halliana*; 4, *M. baccata*; 5, *M. domestica* ‘Golden Delicious’; 6, *M. domestica* ‘Fuji’; 7, *M. domestica* ‘Red Delicious’; 8, *M. domestica* ‘Gala’; and red flower genotypes: 9*, M.* ‘Sparkler’; 10, *M.* ‘Radiant’; 11, *M.* ‘Adams’; 12, *M.* ‘Kelsey’; 13, *M.* ‘Perfect Purple’; 14, *M.* ‘Royalty’. P, phlorizin; T, trilobatin; S, sieboldin.


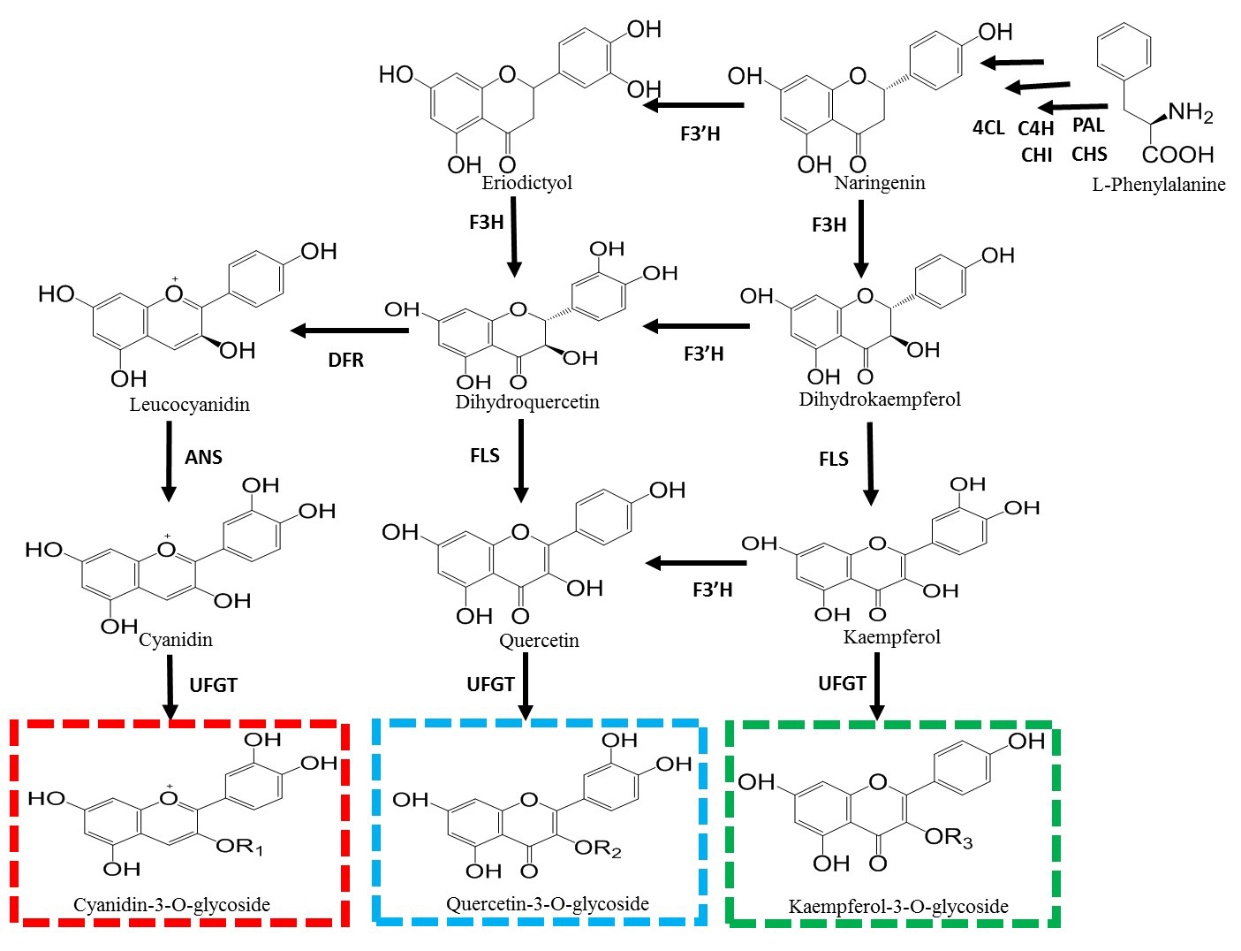


**Fig. S6 Flavonol and anthocyanin synthesis pathway in *Malus*.**


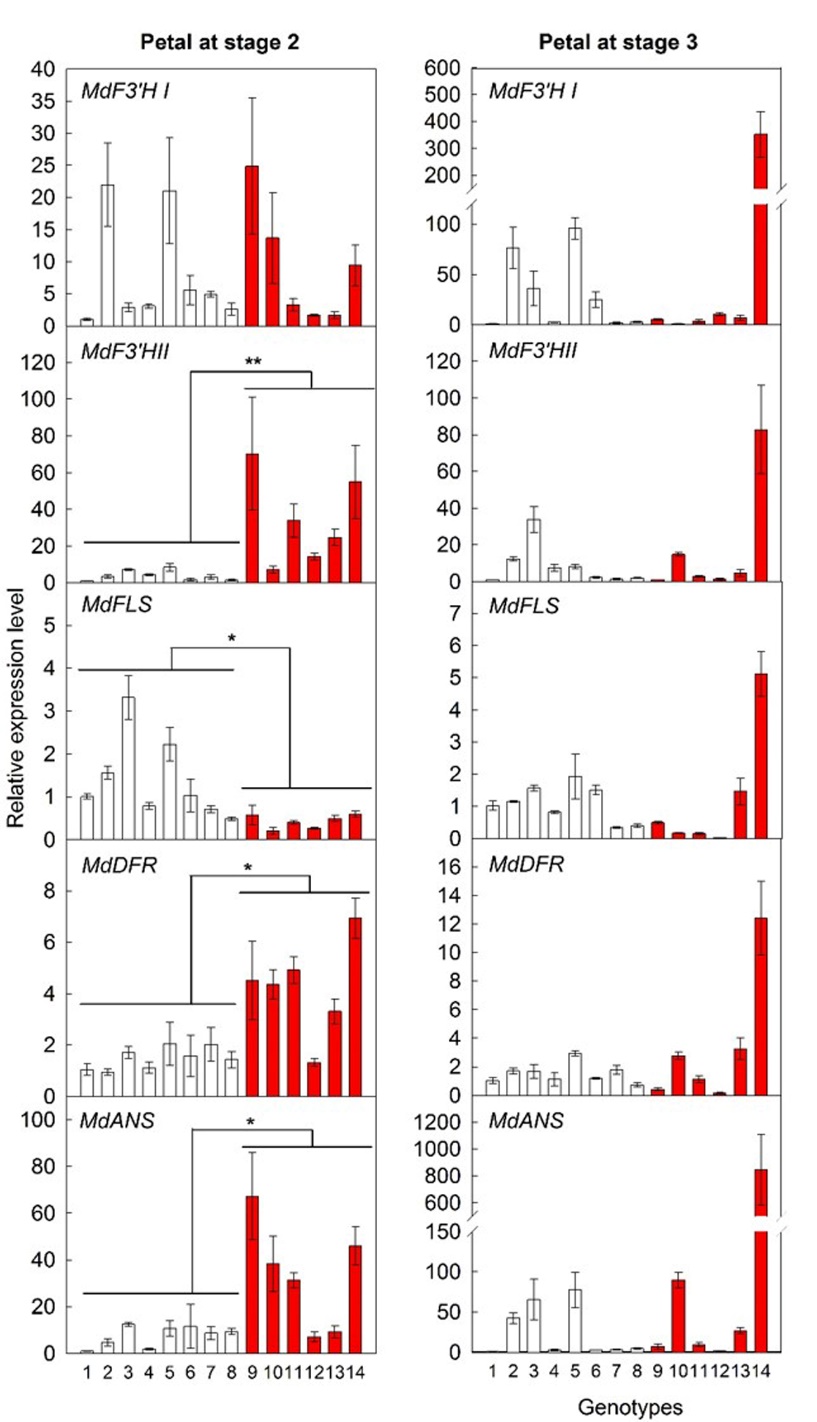


**Fig. S7 Gene expressions in flowers of *Malus* germplasm resources at different stages.**

1, *M. hupehensis*; 2, *M. micromalus*; 3, *M. halliana*; 4, *M. baccata*; 5, *M. domestica* ‘Golden Delicious’; 6, *M. domestica* ‘Fuji’; 7, *M. domestica* ‘Red Delicious’; 8, *M. domestica* ‘Gala’; and red flower genotypes: 9*, M.* ‘Sparkler’; 10, *M.* ‘Radiant’; 11, *M.* ‘Adams’; 12, *M.* ‘Kelsey’; 13, *M.* ‘Perfect Purple’; 14, *M.* ‘Royalty’. F3’H, flavonoid 3’-hydroxylase; FLS, flavonol synthase; DFR dihydroflavonol 4-reductase; ANS, anthocyanin synthase. “**” and “*” mean significant difference between the white flower and the red flower genotypes at P < 0.01 and P < 0.05, respectively, *t*-test. Data are presented as mean ± SE (n = 3).


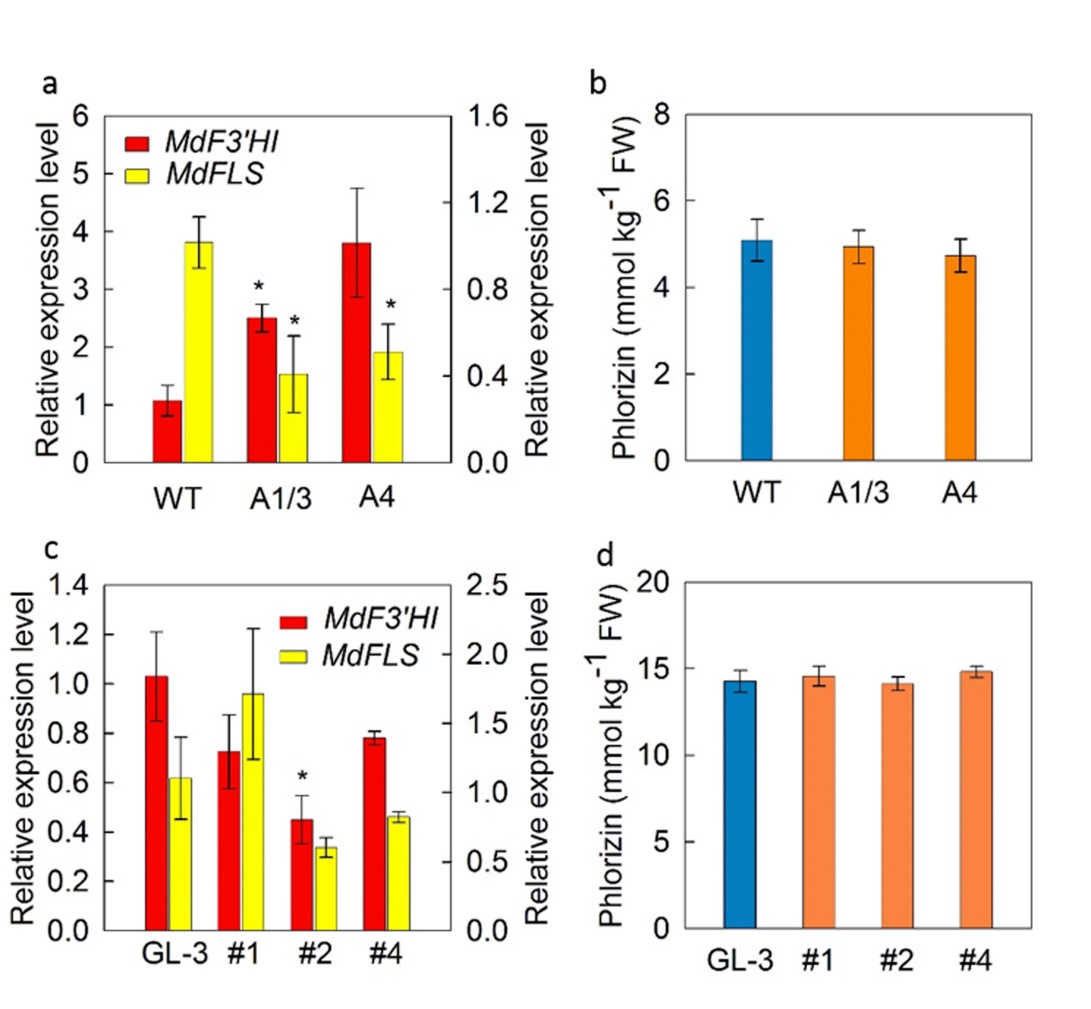


**Fig. S8 The gene expressions and phlorizin concentrations in the flowers of *M. domestica* ‘Royal Gala’ (WT) and its transgenic lines (A1/3, A4) with *MdMYB10* overexpression (a, b) or in the leaves of *M. domestica* ‘GL3’ (GL-3) and its transgenic lines (#1, #2, #4) with the RNAi of *MdF3’HII* (c, d).**

F3’H, flavonoid 3’-hydroxylase; FLS, flavonol synthase. Data are presented as mean ± SE (n = 3 for a and c, n =5 for b and d); “*” means significant difference between the wild type and the transgenic line at P < 0.05, *t*-test.


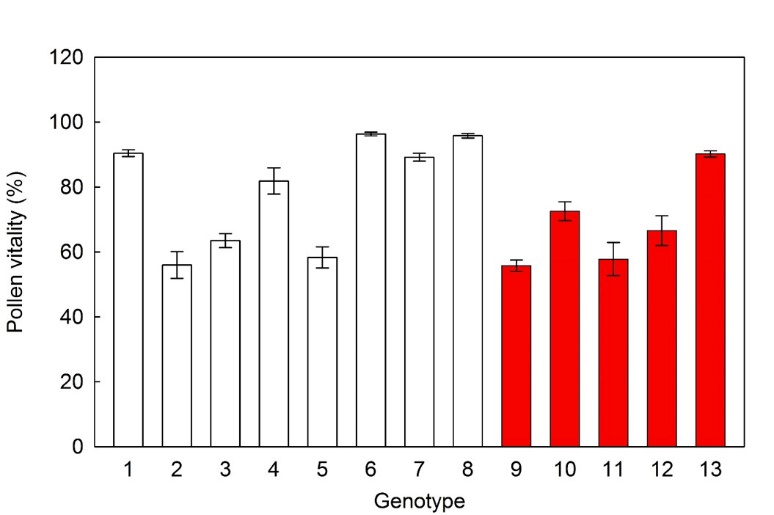


**Fig. S9 Pollen viability of different genotypes of *Malus*.**

1: *M. hupehensis*; 2: *M. micromalus*; 3: *M. halliana*; 4: *M. baccata*; 5: *M. domestica* ‘Golden Delicious’; 6: *M. domestica* ‘Fuji’; 7: *M. domestica* ‘Red Delicious’; 8: *M. domestica* ‘Gala’; 9*: M.* ‘Sparkler’; 10: *M.* ‘Radiant’; 11: *M.* ‘Adams’; 12: *M.* ‘Kelsey’; 13: *M.* ‘Perfect Purple’. Data are presented as mean ± SE (n = 5).


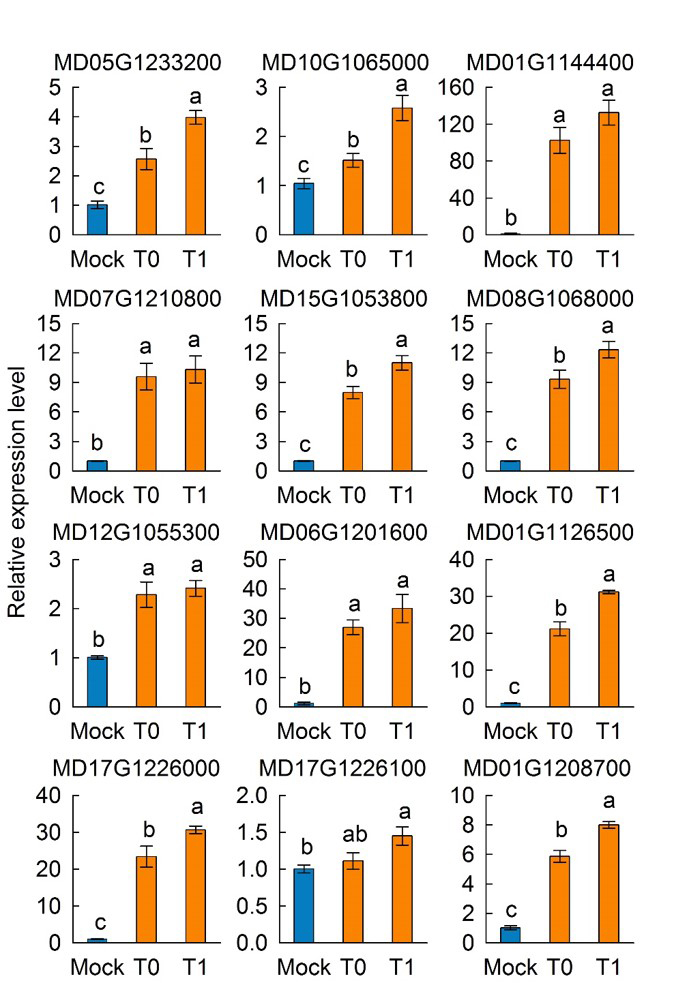


**Fig. S10** **Gene expressions during pollen tube growth double checked by q-PCR.**

Mock: pollens cultivated for 0 hour, T0: pollens cultivated for 4 hours, T1: pollen cultivated for 4 hours with 1 μM kaempferol 3-*O*-rhamnoside treatment. Data are presented as mean ± SE (n = 3). Different letters above the bars indicate statistically significant differences at P < 0.01, LSD.


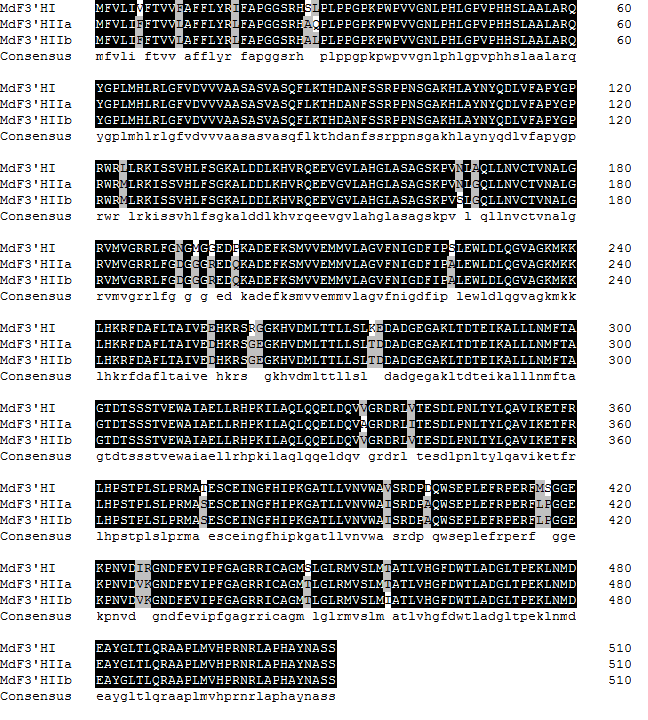


**Fig. S11** **Comparison of amino acid sequences of three F3’H in *Malus.***

The accession numbers for the aligned proteins are: *MdF3’HI*: ACR14867; *MdF3’HIIa*: ACR14868; *MdF3’HIIb*: ACR14869.
