# Supplementary material for: Competition between anthocyanin and kaempferol glycosides biosynthesis affects pollen tube growth and seed set of Malus
Source: Hortic Res. 2021 Aug 1;8:173. doi: 10.1038/s41438-021-00609-9 (PMC8325685; doi:10.1038/s41438-021-00609-9)
Supplement: Supplementary file 2 — Supplementary tables [file 41438_2021_609_MOESM2_ESM.docx]

**Table S1 The NMR-data of three kaempferol glycosides.**

| No. | kaempferol 3-*O*-β-xylopyranoside | | kaempferol 3-*O*-α-arabinofuranoside | | kaempferol 3-*O*-α-rhamnopyranoside | |
| --- | --- | --- | --- | --- | --- | --- |
|  | ^13^C NMR | ^1^H NMR | ^13^C NMR | ^1^H NMR | ^13^C NMR ^1^H NMR | |
| 1 |  |  |  |  |  |  |
| 2 | 156.22 |  | 156.83 |  | 156.57 |  |
| 3 | 133.17 |  | 133.47 |  | 134.29 |  |
| 4 | 177.44 |  | 177.72 |  | 177.79 |  |
| 5 | 161.24 | 12.59 (s, 1H) | 161.25 | 12.63 (s, 1H) | 161.36 | 12.63 (s, 1H) |
| 6 | 98.79 | 6.22 (d, *J* = 2.0 Hz, 1H) | 98.73 | 6.21 (d, *J* = 2.0 Hz, 1H) | 98.80 | 6.22 (d, *J* = 2.0 Hz, 1H) |
| 7 | 164.27 | 10.89 (s, 1H) | 164.26 | 10.88 (s, 1H) | 164.28 | 10.88 (s, 1H) |
| 8 | 93.74 | 6.45 (d, *J* = 2.1 Hz, 1H) | 93.74 | 6.45 (d, *J* = 2.1 Hz, 1H) | 93.82 | 6.42 (d, *J* = 2.0 Hz, 1H) |
| 9 | 156.38 |  | 157.41 |  | 157.31 |  |
| 10 | 103.98 |  | 104.06 |  | 104.22 |  |
| 1’ | 120.73 |  | 120.77 |  | 120.60 |  |
| 2’ | 130.84 | 8.07 – 8.00 (m, 1H) | 130.84 | 8.03 (d, *J* = 8.7 Hz, 1H) | 130.67 | 7.79 – 7.72 (m, 1H) |
| 3’ | 115.31 | 6.96 – 6.87 (m, 1H) | 115.46 | 6.90 (d, *J* = 8.6 Hz, 1H) | 115.47 | 6.95 – 6.88 (m, 1H) |
| 4’ | 160.16 | 10.23 (s, 1H) | 160.00 | 10.19 (s, 1H) | 160.06 | 10.22 (s, 1H) |
| 5’ | 115.31 | 6.96 – 6.87 (m, 1H) | 115.46 | 6.90 (d, *J* = 8.6 Hz, 1H) | 115.47 | 6.95 – 6.88 (m, 1H) |
| 6’ | 130.84 | 8.07 – 8.00 (m, 1H) | 130.84 | 8.03 (d, *J* = 8.7 Hz, 1H) | 130.67 | 7.79 – 7.72 (m, 1H) |
| 1’’ | 101.76 | 5.35 (d, *J* = 7.0 Hz, 1H) | 108.12 | 5.63 (s, 1H) | 101.87 | 5.30 (d, *J* = 1.5 Hz, 1H) |
| 2’’ | 69.46 | 3.24 (m, 1H) | 82.19 | 4.16 (d, *J* = 9.1 Hz, 1H) | 70.69 | 3.19 – 3.04 (m, 1H) |
| 3’’ | 75.86 | 3.24 (m, 1H) | 77.16 | 3.77 – 3.70 (m, 1H) | 70.16 | 4.01 – 3.95 (m, 1H) |
| 4’’ | 73.73 | 3.24 (m, 1H) | 86.40 | 3.54 (q, *J* = 5.1 Hz, 1H) | 70.42 | 3.48 (ddd, *J* = 8.6, 4.9, 3.1 Hz, 1H) |
| 5’’ | 65.99 | 3.63 (dd, *J* = 11.5, 5.1 Hz, 1H) 2.97 (dd, *J* = 11.5, 9.5 Hz, 1H) | 60.92 | 3.28 (m, 2H) | 71.21 | 3.19 – 3.04 (m, 1H) |
| 6’’ |  |  |  |  | 17.54 | 0.80 (d, *J* = 5.9 Hz, 3H) |
| OH-2’’ |  | 5.37 (d, *J* = 4.8 Hz, 1H) |  | 5.50 (d, *J* = 5.4 Hz, 1H) |  | 4.97 (d, *J* = 4.4 Hz, 1H) |
| OH-3’’ |  | 5.08 (d, *J* = 4.5 Hz, 1H) |  | 5.24 (d, *J* = 5.1 Hz, 1H) |  | 4.74 (d, *J* = 4.7 Hz, 1H) |
| OH-4’’ |  | 4.97 (d, *J* = 5.2 Hz, 1H) |  |  |  | 4.64 (d, *J* = 5.8 Hz, 1H) |
| OH-5’’ |  |  |  | 4.64 (t, *J* = 5.1 Hz, 1H) |  |  |

**Table S2.** **Annotation of the differentially expressed genes.**

| Gene_ID | T1_T0 | T0_Mock | T1_Mock | Annotation |
| --- | --- | --- | --- | --- |
|  | Log2 fold change | | |  |
| gene:MD03G1216600 | -0.64803 | -0.37709 | -1.01738 | Transducin/WD40 repeat-like superfamily protein |
| gene:MD00G1078300 | 0.389656 | 0.539484 | 0.939215 | WD40-repeat-containing domain |
| gene:MD11G1039500 | -1.45247 | -1.01405 | -2.46402 | BTB/POZ domain |
| gene:MD03G1273800 | 0.225177 | 7.514709 | 7.749419 | Histone-fold |
| gene:MD02G1134400 | 0.20057 | 7.868851 | 8.0786 | Linker histone H1/H5, domain H15 |
| gene:MD09G1123300 | 0.505541 | 1.169934 | 1.68527 | NAC domain |
| gene:MD01G1144400 | 0.713113 | 5.890922 | 6.61156 | 18.5 KDa class I heat shock protein |
| gene:MD07G1210800 | 0.348292 | 2.483861 | 2.842626 | 16.9 Kda class I heat shock protein 2 |
| gene:MD15G1053800 | 0.306008 | 2.304464 | 2.620538 | 17.1 KDa class II heat shock protein |
| gene:MD08G1068000 | 0.61813 | 6.431353 | 7.05625 | 17.3 Kda class II heat shock protein |
| gene:MD17G1226000 | 0.42678 | 6.332054 | 6.771213 | Heat shock protein 70 family |
| gene:MD17G1226100 | 0.325438 | 3.52406 | 3.861386 | Heat shock protein 70 family |
| gene:MD01G1126500 | 0.276233 | 3.407836 | 3.693972 | Heat shock protein 70 family |
| gene:MD01G1208700 | 0.253973 | 0.662921 | 0.927164 | Heat shock protein Hsp90 family |
| gene:MD12G1055300 | 0.519256 | 2.661967 | 3.191192 | Activator of Hsp90 ATPase, N-terminal |
| gene:MD00G1151100 | 0.705178 | 1.004307 | 1.722338 | Serine-threonine/tyrosine-protein kinase, catalytic domain |
| gene:MD04G1187300 | 0.649105 | 1.421967 | 2.081676 | serine/threonine-protein kinase haspin homolog |
| gene:MD05G1233200 | 0.628056 | 0.725112 | 1.362934 | Dedicator of cytokinesis C-terminal |
| gene:MD10G1065000 | 0.674697 | 0.899021 | 1.584826 | Rab escort protein |
| gene:MD05G1062600 | -0.21193 | -0.24915 | -0.45178 | Sec14p-like phosphatidylinositol transfer family protein |
| gene:MD04G1176700 | 0.280906 | 2.502598 | 2.793348 | mucin-5AC-like |
| gene:MD06G1201600 | 0.380002 | 6.194136 | 6.582805 | ATPase, AAA-type, core |
| gene:MD06G1067900 | 1.47654 | 2.688926 | 4.178138 | Kinesin motor domain |
| gene:MD08G1078700 | 0.345676 | 1.681181 | 2.036075 | Kinesin motor domain |
| gene:MD14G1216600 | 0.288004 | 1.361346 | 1.659516 | movement protein binding protein 2C Microtubule-associated and viral movement protein binding protein |
| gene:MD03G1268600 | 0.439356 | 1.611757 | 2.06148 | protein HUA2 |
| gene:MD13G1197300 | 0.515541 | 1.404608 | 1.927067 | Pentatricopeptide repeat (PPR) superfamily protein |
| gene:MD15G1002100 | 0.235113 | 0.81772 | 1.062736 | Pentatricopeptide repeat (PPR-like) superfamily protein |
| gene:MD15G1329400 | -0.61428 | -0.7455 | -1.34831 | Mitochondrial inner membrane protein Mitofilin |
| gene:MD09G1016800 | 0.566951 | 0.688052 | 1.264312 | prohibitin-3, mitochondrial-like |
| gene:MD00G1161000 | 0.656557 | 2.601736 | 3.267121 | Synaptotagmin-like mitochondrial-lipid-binding domain |
| gene:MD08G1047300 | -0.32815 | -0.34921 | -0.66653 | Peptidase C19, ubiquitin carboxyl-terminal hydrolase |
| gene:MD13G1214800 | -1.16196 | -0.89175 | -2.02572 | putative E3 ubiquitin-protein ligase XBAT35 |
| gene:MD10G1182800 | -0.24063 | -0.45791 | -0.68837 | Sugar isomerase (SIS) |
| gene:MD17G1157900 | -0.32703 | -0.85972 | -1.1784 | Major facilitator, sugar transporter-like |
| gene:MD13G1148300 | 0.228745 | 0.345962 | 0.584589 | Glycosyl hydrolase family 100 |
| gene:MD00G1076100 | 0.349503 | 2.65576 | 3.015138 | glycolipid transfer protein 1 |
| gene:MD16G1221400 | 0.858017 | 2.93493 | 3.81926 | Glycolipid transfer protein domain |
| gene:MD12G1100900 | 0.643224 | 1.766775 | 2.419957 | Transmembrane protein TauE-like |
| gene:MD02G1190300 | -0.29844 | -0.29844 | -0.58738 | Squalene epoxidase |
| gene:MD05G1259800 | 0.652573 | 1.964566 | 2.626317 | - |

**Table S3.** **Primers used for qRT-PCR.**

| Genes Name/ID | Forward primer (5’ to 3’) |  | Reverse primer (5’ to 3’) |
| --- | --- | --- | --- |
| *Actin* | TGACCGAATGAGCAAGGAAATTACT |  | TACTCAGCTTTGGCAATCCACATC |
| *F3’HI* | ATGTCGGGAGGTGAGAAGCCCAATGTC |  | CGGCTCTTTGTAGGGTGAGCCCATAG |
| *F3’HII* | CAGAAGGCCGACGAGTTCAAATCC |  | ATGTCCACGTGCTTCCCTTCG |
| *FLS* | CTTCTTACAGGGAAGCTAATGAA |  | GAGGACATGGTGGGTAGTAGT |
| *DFR* | GATAGGGTTTGAGTTCAAGTA |  | TCTCCTCAGCAGCCTCAGTTTTCT |
| *ANS* | CCAAGTGAAGCGGGTTGTGCT |  | CAAAGCAGGCGGACAGGAGTAGC |
| MD08G1068000 | GACGCCGAGAAGTCCAACGCC |  | TTACCGACCCTCCGCTCCATC |
| MD07G1210800 | CACCACCCGTTCCGCTCACTCT |  | CGTCCTCCACCTCAACCTTCACC |
| MD15G1053800 | CCTAACTCCTACGTGTTCGTGGTG |  | CGCATCTACATTCGCATTCTCC |
| MD12G1055300 | AATCAGCCTCAATCTGTCGTG |  | CCGCCCTTAGCCATACTCT |
| MD06G1201600 | GGTTCCAGCAGGTTTACGT |  | GATGTCGCCCTGTGATGTAC |
| MD01G1208700 | CTGACAAGGCTAACAAGACCC |  | AGAAACCGACACCAAACTGA |
| MD01G1126500 | AGCGGTGAGGGTAACGAGA |  | CCAGGCTGATTGTCTGAGTA |
| MD17G1226100 | AACTCGCTTGAGAACTACGC |  | CCAGGACGCTGATACATTTT |
| MD17G1226000 | CAAGAGCATAAACCCTGACG |  | TGTTTCTCGGAATCAAGACG |
| MD05G1233200 | AGATTTGGGAAGCAGAAGG |  | CAAGAGCTGGGAATGAACC |
| MD10G1065000 | AAGATGATAAAGGAAAGGTGGCT |  | CATGGCGGCATGTAGTGAT |
| MD01G1144400 | ACCCAACTCTAATCCGGCATCT |  | CCGTCCTCCACCTCCACTT |

**Table S4. Statistical analysis results.**

| Items | Comparison | F values | P values | Remarks |
| --- | --- | --- | --- | --- |
| Seed numbers | WF vs RF | 12.605 | 0.000 | Figure 1C |
| Seed numbers | WT vs A1/3 | 0.356 | 0.014 | Figure 1D |
| Cyanidin 3-*O*-glycosides in petal | WF vs RF | 6.46 | 0.000 | Figure 3 |
| Cyanidin 3-*O*-glycosides in pistil | WF vs RF | 6.132 | 0.001 | Figure 3 |
| Cyanidin 3-*O*-glycosides in stamen | WF vs RF | 10.268 | 0.000 | Figure 3 |
| Cyanidin 3-*O*-glycosides in leaf | WF vs RF | 7.879 | 0.056 | Figure 3 |
| Cyanidin 3-*O*-glycosides in fruit | WF vs RF | 15.441 | 0.000 | Figure 3 |
| Kaempferol 3-O-glycosides in petal | WF vs RF | 34.909 | 0.000 | Figure 3 |
| Kaempferol 3-O-glycosides in pistil | WF vs RF | 9.982 | 0.028 | Figure 3 |
| Kaempferol 3-O-glycosides in stamen | WF vs RF | 12.075 | 0.000 | Figure 3 |
| Kaempferol 3-O-glycosides in leaf | WF vs RF | 1.752 | 0.153 | Figure 3 |
| Kaempferol 3-O-glycosides in fruit | WF vs RF | nt | nt | Figure 3 |
| Quercetin 3-O-glycosides in petal | WF vs RF | 0.089 | 0.031 | Figure 3 |
| Quercetin 3-O-glycosides in pistil | WF vs RF | 4.823 | 0.003 | Figure 3 |
| Quercetin 3-O-glycosides in stamen | WF vs RF | 0.14 | 0.213 | Figure 3 |
| Quercetin 3-O-glycosides in leaf | WF vs RF | 0.203 | 0.036 | Figure 3 |
| Quercetin 3-O-glycosides in fruit | WF vs RF | 2.734 | 0.536 | Figure 3 |
| *MdF3’HI* in flower | WF vs RF | 2.964 | 0.487 | Figure 4 |
| *MdF3’HI* in leaf | WF vs RF | 3.368 | 0.176 | Figure 4 |
| *MdF3’HI* in fruit | WF vs RF | 0.473 | 0.817 | Figure 4 |
| *MdF3’HII* in flower | WF vs RF | 0.005 | 0.000 | Figure 4 |
| *MdF3’HII* in leaf | WF vs RF | 1.321 | 0.078 | Figure 4 |
| *MdF3’HII* in fruit | WF vs RF | 0.001 | 0.289 | Figure 4 |
| *MdFLS* in flower | WF vs RF | 2.701 | 0.000 | Figure 4 |
| *MdFLS* in leaf | WF vs RF | 3.902 | 0.016 | Figure 4 |
| *MdFLS* in fruit | WF vs RF | 16.136 | 0.167 | Figure 4 |
| *MdDFR* in flower | WF vs RF | 1.077 | 0.138 | Figure 4 |
| *MdDFR* in leaf | WF vs RF | 10.032 | 0.235 | Figure 4 |
| *MdDFR* in fruit | WF vs RF | 6.133 | 0.457 | Figure 4 |
| *MdANS* in flower | WF vs RF | 30.274 | 0.011 | Figure 4 |
| *MdANS* in leaf | WF vs RF | 4.217 | 0.164 | Figure 4 |
| *MdSNS* in fruit | WF vs RF | 0.141 | 0.772 | Figure 4 |
| *MdF3’HII* | WT vs A1/3 | 6.013 | 0.006 | Figure 5B |
| *MdF3’HII* | WT vs A4 | 10.361 | 0.004 | Figure 5B |
| Kaempferol 3-O-glycosides | WT vs A1/3 | 13.519 | 0.000 | Figure 5C |
| Kaempferol 3-O-glycosides | WT vs A4 | 13.603 | 0.000 | Figure 5C |
| Cyanidin 3-O-glycosides | WT vs A1/3 | 8.391 | 0.001 | Figure 5C |
| Cyanidin 3-O-glycosides | WT vs A4 | 8.946 | 0.000 | Figure 5C |
| Quercetin 3-O-glycosides | WT vs A1/3 | 4.239 | 0.008 | Figure 5C |
| Quercetin 3-O-glycosides | WT vs A4 | 7.963 | 0.003 | Figure 5C |
| *MdF3’HII* | GL-3 vs #1 | 7.562 | 0.008 | Figure 5E |
| *MdF3’HII* | GL-3 vs #2 | 2.119 | 0.009 | Figure 5E |
| *MdF3’HII* | GL-3 vs #4 | 0.452 | 0.04 | Figure 5E |
| Kaempferol 3-O-glycosides | GL-3 vs #1 | 6.724 | 0.000 | Figure 5F |
| Kaempferol 3-O-glycosides | GL-3 vs #2 | 1.161 | 0.000 | Figure 5F |
| Kaempferol 3-O-glycosides | GL-3 vs #4 | 0.575 | 0.403 | Figure 5F |
| Quercetin 3-O-glycosides | GL-3 vs #1 | 0.352 | 0.217 | Figure 5F |
| Quercetin 3-O-glycosides | GL-3 vs #2 | 0.508 | 0.872 | Figure 5F |
| Quercetin 3-O-glycosides | GL-3 vs #4 | 3.696 | 0.391 | Figure 5F |
| Seed numbers (*M.* ‘Radiant’) | Mock vs 50 μM K3 | 1.358 | 0.004 | Figure 8B |
| Seed numbers (*M.* ‘Radiant’) | Mock vs 100 μM K3 | 2.145 | 0.006 | Figure 8B |
| Seed numbers (*M.* ‘Adams’) | Mock vs 50 μM K3 | 1.635 | 0.005 | Figure 8B |
| Seed numbers (*M.* ‘Adams’) | Mock vs 100 μM K3 | 1.365 | 0.007 | Figure 8B |
| Seed numbers (*M.* *hallinana*) | Mock vs 50 μM C1 | 1.537 | 0.361 | Figure 8B |
| Seed numbers (*M.* *hallinana*) | Mock vs 100 μM C1 | 0.869 | 0.372 | Figure 8B |
| Seed numbers (*M.* *micromalus*) | Mock vs 50 μM C1 | 1.262 | 0.247 | Figure 8B |
| Seed numbers (*M.* *micromalus*) | Mock vs 100 μM C1 | 0.158 | 0.47 | Figure 8B |
| Cyanidin 3-*O*-glycosides in petal at stage 2 | WF vs RF | 10.104 | 0.000 | Supplemental Figure S4 |
| Kaempferol 3-*O*-glycosides in petal at stage 2 | WF vs RF | 19.753 | 0.000 | Supplemental Figure S4 |
| Quercetin 3-*O*-glycosides in petal at stage 2 | WF vs RF | 0.742 | 0.013 | Supplemental Figure S4 |
| Cyanidin 3-*O*-glycosides in petal at stage 3 | WF vs RF | 5.803 | 0.000 | Supplemental Figure S4 |
| Kaempferol 3-*O*-glycosides in petal at stage 3 | WF vs RF | 12.346 | 0.001 | Supplemental Figure S4 |
| Quercetin 3-*O*-glycosides in petal at stage 3 | WF vs RF | 0.9 | 0.001 | Supplemental Figure S4 |
| Dihydrochalcones in petal at stage 1 | WF vs RF | 0.181 | 0.736 | Supplemental Figure S5 |
| Dihydrochalcones in petal at stage 2 | WF vs RF | 0.011 | 0.679 | Supplemental Figure S5 |
| Dihydrochalcones in petal at stage 3 | WF vs RF | 0.013 | 0.788 | Supplemental Figure S5 |
| Dihydrochalcones in pistil | WF vs RF | 0.055 | 0.669 | Supplemental Figure S5 |
| Dihydrochalcones in stamen | WF vs RF | 4.726 | 0.243 | Supplemental Figure S5 |
| Dihydrochalcones in leaf | WF vs RF | 1.677 | 0.16 | Supplemental Figure S5 |
| Dihydrochalcones in fruit | WF vs RF | 0.799 | 0.447 | Supplemental Figure S5 |
| *MdF3’HI* in flower at stage 2 | WF vs RF | 0.001 | 0.804 | Supplemental Figure S7 |
| *MdF3’HII* in flower at stage 2 | WF vs RF | 14.282 | 0.004 | Supplemental Figure S7 |
| *MdFLS* in flower at stage 2 | WF vs RF | 7.113 | 0.031 | Supplemental Figure S7 |
| *MdDFR* in flower at stage 2 | WF vs RF | 4.451 | 0.002 | Supplemental Figure S7 |
| *MdANS* in flower at stage 2 | WF vs RF | 9.099 | 0.011 | Supplemental Figure S7 |
| *MdF3’HI* in flower at stage 3 | WF vs RF | 3.903 | 0.532 | Supplemental Figure S7 |
| *MdF3’HII* in flower at stage 3 | WF vs RF | 3.034 | 0.455 | Supplemental Figure S7 |
| *MdFLS* in flower at stage 3 | WF vs RF | 3.945 | 0.844 | Supplemental Figure S7 |
| *MdDFR* in flower at stage 3 | WF vs RF | 4.929 | 0.283 | Supplemental Figure S7 |
| *MdANS* in flower at stage 3 | WF vs RF | 6.451 | 0.263 | Supplemental Figure S7 |
| *MdF3’HI* | WT vs A1/3 | 0.001 | 0.016 | Supplemental Figure S8A |
| *MdF3’HI* | WT vs A4 | 3.299 | 0.051 | Supplemental Figure S8A |
| *MdFLS* | WT vs A1/3 | 1.027 | 0.047 | Supplemental Figure S8A |
| *MdFLS* | WT vs A4 | 0.001 | 0.045 | Supplemental Figure S8A |
| Phlorizin | WT vs A1/3 | 0.326 | 0.817 | Supplemental Figure S8B |
| Phlorizin | WT vs A4 | 8.437 | 0.059 | Supplemental Figure S8B |
| *MdF3’HI* | GL-3 vs #1 | 0.26 | 0.263 | Supplemental Figure S8C |
| *MdF3’HI* | GL-3 vs #2 | 2.406 | 0.047 | Supplemental Figure S8C |
| *MdF3’HI* | GL-3 vs #4 | 10.554 | 0.243 | Supplemental Figure S8C |
| *MdFLS* | GL-3 vs #1 | 1.585 | 0.337 | Supplemental Figure S8C |
| *MdFLS* | GL-3 vs #2 | 7.876 | 0.059 | Supplemental Figure S8C |
| *MdFLS* | GL-3 vs #4 | 10.716 | 0.398 | Supplemental Figure S8C |
| Phlorizin | GL-3 vs #1 | 0.049 | 0.744 | Supplemental Figure S8D |
| Phlorizin | GL-3 vs #2 | 0.008 | 0.43 | Supplemental Figure S8D |
| Phlorizin | GL-3 vs #4 | 2.694 | 0.48 | Supplemental Figure S8D |
| Pollen viability | WT vs RT | 0.169 | 0.722 | Supplemental Figure S9 |
